# Unveiling the Role of Capping Groups in Naphthalene N-Capped Dehydrodipeptide Hydrogels

**DOI:** 10.3390/gels9060464

**Published:** 2023-06-06

**Authors:** Helena Vilaça, André Carvalho, Tarsila Castro, Elisabete M. S. Castanheira, Loic Hilliou, Ian Hamley, Manuel Melle-Franco, Paula M. T. Ferreira, José A. Martins

**Affiliations:** 1 Centre of Chemistry, University of Minho, Campus de Gualtar, 4710-057 Braga, Portugal; hvilaca@citeve.pt (H.V.); andrefcarvalho95@gmail.com (A.C.); 2 Department of Chemistry and Biotechnology, Technological Centre for the Textile and Clothing Industries of Portugal, 4760-034 Vila Nova de Famalicão, Portugal; 3 Centre of Biological Engineering, University of Minho, Campus de Gualtar, 4710-057 Braga, Portugal; castro.tarsila@ceb.uminho.pt; 4 Physics Centre of Minho and Porto Universities (CF-UM-UP), University of Minho, Campus de Gualtar, 4710-057 Braga, Portugal; ecoutinho@fisica.uminho.pt; 5 Institute for Polymers and Composites, Department of Polymer Engineering, University of Minho, Campus de Azurém, 4800-058 Guimarães, Portugal; loic@dep.uminho.pt; 6 Department of Chemistry, University of Reading, Whiteknights, P.O. Box 224, Reading RG6 6AD, UK; i.w.hamley@reading.ac.uk; 7 CICECO-Aveiro Institute of Materials, Department of Chemistry, University of Aveiro, 3810-193 Aveiro, Portugal; manuelmelle.research@gmail.com

**Keywords:** dehydrodipeptide, 1-naphthyl group, 2-naphthylacetyl group, self-assembly, hydrogel

## Abstract

Self-assembled peptide-based hydrogels are archetypical nanostructured materials with a plethora of foreseeable applications in nanomedicine and as biomaterials. N-protected di- and tri-peptides are effective minimalist (molecular) hydrogelators. Independent variation of the capping group, peptide sequence and side chain modifications allows a wide chemical space to be explored and hydrogel properties to be tuned. In this work, we report the synthesis of a focused library of dehydrodipeptides N-protected with 1-naphthoyl and 2-naphthylacetyl groups. The 2-naphthylacetyl group was extensively reported for preparation of peptide-based self-assembled hydrogels, whereas the 1-naphthaloyl group was largely overlooked, owing presumably to the lack of a methylene linker between the naphthalene aromatic ring and the peptide backbone. Interestingly, dehydrodipeptides N-capped with the 1-naphthyl moiety afford stronger gels, at lower concentrations, than the 2-naphthylacetyl-capped dehydrodipeptides. Fluorescence and circular dichroism spectroscopy showed that the self-assembly of the dehydrodipeptides is driven by intermolecular aromatic π–π stacking interactions. Molecular dynamics simulations revealed that the 1-naphthoyl group allows higher order aromatic π–π stacking of the peptide molecules than the 2-naphthylacetyl group, together with hydrogen bonding of the peptide scaffold. The nanostructure of the gel networks was studied by TEM and STEM microscopy and was found to correlate well with the elasticity of the gels. This study contributes to understanding the interplay between peptide and capping group structure on the formation of self-assembled low-molecular-weight peptide hydrogels. Moreover, the results presented here add the 1-naphthoyl group to the palette of capping groups available for the preparation of efficacious low-molecular-weight peptide-based hydrogels.

## 1. Introduction

The nanotechnology revolution is underway with a foreseeable unprecedented impact on science, technology and society [1]. Novel functional materials, nanostructured at different organization levels, embody one of the main achievements of nanotechnology [2]. The surge of advancements in nanomedicine and biomaterials research illustrates the essential role of nanotechnology in science and technology and its predictable impact in society [3,4,5,6,7]. Hydrogels, viscoelastic solid-like materials with high water content, generally above 99%, are archetypical biomaterials: high water content, biocompatibility, tunable structure, function and responsiveness to external stimuli. In hydrogels, water molecules are trapped by a three-dimensional network of fibers made of gelator molecules [8]. Macromolecular gelators—synthetic and natural polymers—originate hydrogels via physical (reversible) and/or chemical (irreversible) crosslinking of long oligomer chains [9]. Molecular gelators—low-molecular-weight natural and synthetic compounds (peptides, oligosaccharides, nucleotides, and others)—form self-assembled (supramolecular) hydrogels through hierarchal bottom-up processes: self-assembly of gelator molecules into fiber-like nanostructures (nanotubes, tapes, ribbons, etc.) followed by their physical entanglement. The self-assembly of low-molecular-weight gelators (LMWG) is driven by an ensemble of weak non-covalent interactions: hydrophobic and aromatic π–π stacking interactions, electrostatic and hydrogen bonding [10,11]. The formation of self-assembled (supramolecular) hydrogels is a process reminiscent of the fibrillation of peptides and proteins in vitro and in vivo into amyloid structures [12]. Logically, peptides are ideal low-molecular-weight gelators (LMWG)–a variety of available amino acid building blocks, amenable to automated (solid-phase) synthesis and structural modification, which together allow the nanofibrillar structure and macroscopic properties of hydrogels to be tuned to specific functions [13,14]. Peptide-based supramolecular hydrogels are generally biocompatible, biodegradable and responsive to environmental stimuli owing to their self-assembled nature. Naturally, a wide range of applications have been devised for self-assembled peptide-based hydrogels: drug delivery, biosensors, wound healing, matrixes for 3D cell culture, regenerative medicine and others [15,16]. Importantly, the structural and functional versatility of peptide-based supramolecular hydrogels can be augmented by incorporation of drugs, biomolecules, nanoparticles, SPION, graphene oxide, carbon nanotubes and others, which add new properties and functional capabilities to hydrogels, e.g., magnetism and paramagnetism, hyperthermia, etc. [17,18,19,20]. The gelation of low-molecular-weight peptides is likely to be the result of a subtle balance between solubility and aggregation kinetics in water. Thus, low-molecular-weight peptide hydrogelators usually contain both hydrophobic and hydrophilic amino acids [10,11]. Specific peptide sequences (motifs) display high gelation propensity owing to self-assembly into cross β-sheet structures [21,22]. Although aromatic amino acids (Phe, Tyr, Trp), able to engage in π–π stacking interactions, are not absolutely required for gelation, generally a higher content of aromatic amino acid residues correlates with the formation of stronger (elastic) gels and determines the preference for specific arrangements of the peptides into fibers [23]. Generally, peptides exhibiting higher hydrophobicity afford gels at lower critical gelation concentrations (CGC) and higher pH values [24]. Protected amino acids and di- and tri-peptides containing aromatic amino-acid residues (Phe, Tyr, Trp) and aromatic N-protecting groups, e.g., Fmoc, indole, or naphthalene derivatives, are ultra-small minimalist gelators [10,11,12,13,14,25]. Despite some empirical rules on the design of peptide sequences prone to self-assembly, it is still difficult to predict how peptide self-assembly determines the size (diameter and length) and morphology (nanotubes, ribbons, tapes, and others) of the fibers and the ensuing 3D architecture of the fiber network [26]. Our research group reported hydrogels made of dehydrodipeptides N-conjugated to the naphthalene-derived drug naproxen—a non-steroidal anti-inflammatory (NSAID) drug [27,28,29]. The naproxen-capped hydrogelators were biocompatible and not only retain the NSAID properties, but exhibit novel pharmacological properties—as LOX inhibitors, not related to naproxen [30]. Moreover, the non-canonical dehydroamino acids (dehydrophenylalanine—ΔPhe, dehydroalanine—ΔAla and dehydroaminobutyric acid—ΔAbu) endow the hydrogelator molecules (and the hydrogels) with proteolytic resistance, which is important to ensure a suitable life-time for the hydrogels in in vivo applications [29]. In this work, we prepared a focused library of dehydrodipeptides N-capped with the naphthalene-derived protecting groups 2-naphthylacetyl (2-Naph) and 1-naphthoyl (1-Nap) to get insight into the interplay between peptide composition and capping group structure on the self-assembly and nanostructure of the hydrogels and the rheological properties. Self-assembly of the hydrogels was studied by fluorescence spectroscopy, circular dichroism (CD), transmission electron microscopy (TEM), and molecular dynamics (MD) simulations. Rheology studies were performed to evaluate the mechanical properties of the hydrogels.

## 2. Results and Discussion

### 2.1. Synthesis of N-Protected Dehydrodipeptides

**Figure 1:**
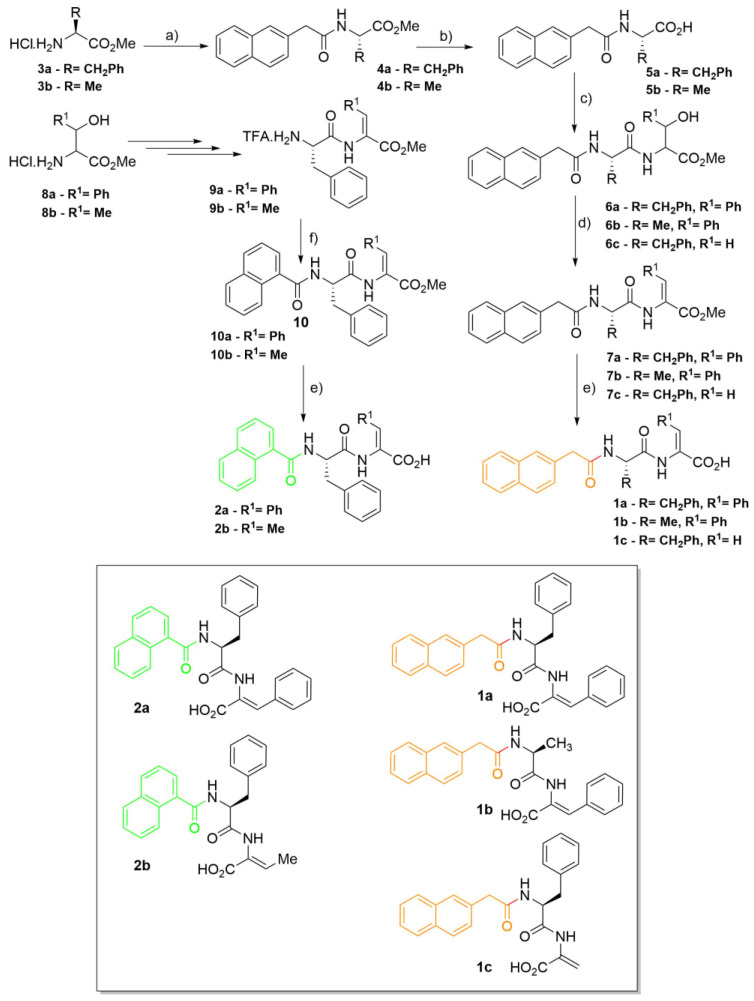
Synthetic pathway for dehydrodipeptides N-protected with 2-naphthylacetyl (2-Naph; **1a**–**c**) and 1-naphthaloyl (1-Nap; **2a**,**b**) groups: (a) 2-(naphthalene-2-yl)acetic acid, DCC/HOBt, NEt_3_, ACN, rt; (b) i. NaOH (1 M), methanol, rt, ii. HCl (1 M); (c) phenylserine methyl ester hydrochloride (R^1^ = Ph), threonine methyl ester hydrochloride (R^1^ = Me), serine methyl ester hydrochloride (R^1^ = H), DCC/HOBt, NEt_3_, ACN, rt; (d) i. Boc_2_O, DMAP, dry ACN, rt, ii. TMG; (e) i. NaOH (1 M), 1,4-dioxane, rt, ii. KHSO_4_ (1 M). Synthesis of 1-naphthaloyldehydrodipeptides **2a** and **2b**: (f) NEt_3_, 1-naphthaloylchloride, DCM, rt.

A focused library of dehydrodipeptides N-capped with naphthalene-derived carboxylic acid groups: 2-naphthylacetyl (2-(naphthalen-2-yl)acetyl) (2-Naph; **1a**–**c**) and 1-naphthaloyl (1-Nap; **2a** and **2b**) was prepared following synthetic methodologies developed by the research group (Figure 1) [27,28,29,31].

For the synthesis of the 2-Naph-protected dehydropeptides (**1a**–**c**), the aromatic capping group was first installed in the N-terminal amino acid (Phe or Ala) using standard carbodiimide coupling conditions. The N,C orthogonally protected amino acids (**4**) were C-deprotected under alkaline conditions to make the carboxylic acid group available for amide coupling to β-hydroxy amino acid methyl esters (phenylserine methyl ester or serine methyl ester). The protected dipeptides (**6**) were subjected to dehydration by a two-step one-pot procedure starting by preparing in situ carbonate esters by treating the β-hydroxy dipeptides with di-*tert*-butyl dicarbonate and 4-dimethylaminopyridine (Boc_2_O/DMAP) followed by CO_2_/*tert*-BuOH elimination upon treatment with tetramethylguanidine (TMG). Saponification afforded the C-deprotected 2-Naph N-capped dehydrodipeptides (**1a**–**c**). The synthesis of the 1-naphthaloyl (1-Nap) N-capped dehydrodipeptides (**2a**,**b**) was attained by reversing the order of the N-capping and dehydration steps: Boc-protected dipeptides containing C-terminal β-hydroxyamino acids (**9**) were first synthesized, followed by dehydration, Boc deprotection and installation of the protecting group. Saponification afforded the 1-Nap N-capped dehydropeptides (**2a**,**b**).

Installing first the N-capping group allows straightforward variation of the dehydroamino acid residue, whereas installing the protecting group at the end of the synthesis allows easy diversification of the N-capping group. Combining both strategies gives expedited access to focused libraries of N-capped dehydropeptide-based hydrogelators.

The peptide series **1a**–**c** and **2a**,**b** allow us to infer the effect of peptide composition, aromaticity (content of aromatic amino acid residues) and hydrophobicity on the self-assembly and rheological properties of the hydrogels. Direct comparison of hydrogelators **1a** and **2a**, sharing the peptide sequence, can reveal the effect of the structure of the N-capping group.

### 2.2. Preparation of Hydrogels

**Figure 2:**
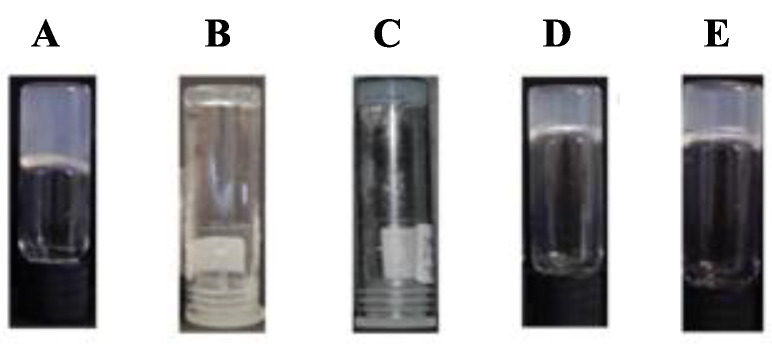
Optical images of hydrogels of dehydrodipeptides: (**A**) **1a** (0.14 wt%, phosphate buffer 0.1 M, pH 8.0); (**B**) **1b** (0.5 wt%, GDL, pH 6.0); (**C**) **1c** (0.6 wt%, GDL, pH 4.0); (**D**) **2a** (0.07 wt%, GDL, pH 5.0); (**E**) **2b** (0.2 wt%, GDL, pH 5.0).

Hydrogelators **1b**, **1c**, **2a** and **2b** revealed very low solubility in phosphate buffer (0.1 M, pH 8.0). Dehydrodipeptide **1a** afforded hydrogels upon cooling (on standing) to r.t. hot solutions (circa 80 °C) in phosphate buffer (0.1 M, pH 8.0). Dehydrodipeptides **1a**–**c** and **2a**,**b** could be made soluble in water upon adjustment to pH circa 10 with aqueous NaOH (1 M). Hydrogel formation was triggered by slow pH dropping by hydrolysis of added D-glucono-δ-lactone (GDL). As the hydrogelator molecules have an ionizable C-terminal carboxylic acid, the hydrogelator’s charge is pH-dependent. Therefore, the self-assembly, nanostructure and macroscopic properties of the hydrogels can be determined by the pH of the hydrogel, which is in turn determined by the molar ratio of GDL/NaOH and by the hydrogelator concentration. Phase diagrams, relating the macroscopic outcome of gelation experiments (gel, suspension, precipitate, solution) with the hydrogel’s pH and the ratio GDL/NaOH (at fixed hydrogelator concentrations) and vice versa, were constructed for hydrogelators **1b**,**c** and **2a**,**b** (Supplementary Materials, Figures S1–S3). The optimal gelation conditions for dehydrodipeptides **1a**–**c** and **2a**,**b** are summarized in Table 1. Photographs of the hydrogels are shown in Figure 2.

It is noteworthy that hydrogelator **1a**, displaying the highest hydrophobicity within this peptide series, is soluble in hot phosphate buffer pH 8 and affords a hydrogel at low concentration (0.14 wt%) upon cooling on standing to room temperature. Regarding GDL triggered-gelation, the CGC is determined by the interplay between hydrogelator hydrophobicity, peptide structure (aromaticity) and N-capping group. Hydrogelators **1a** and **1b** afforded hydrogels at the same concentration and pH (0.5 wt%, pH 6). Higher concentration and a lower hydrogel pH are required for gelation of peptide **1c** (0.6 wt%, pH 4) compared to hydrogelator **1a**, likely due to lower hydrophobicity and aromaticity. As peptides **1b** and **1c** (constitutional isomers) display similar hydrophobicity and aromaticity, it seems that the terminal ∆Phe residue plays a determining role in the self-assembly of the 2-Naph-capped hydrogelators. The most striking result is the exceptionally low CGC of peptide **2a** (0.07 wt%, pH 5; 0.2 wt%, pH 6), capped with the 1-Nap group, in comparison to the same 2-Naph-protected dehydrodipeptide **1a** (0.5 wt%; pH 6). Peptide **2b**, structurally analogous to **1c**, and with similar hydrophobicity, is also a much more effective gelator (0.2 wt%, pH 6) than the 2-Naph-protected peptide **1c**. By comparing the CGC of hydrogelators **2a** and **2b**, it is clear that the higher hydrophobicity/aromaticity of peptide **2a** is also decisive for gelation. As a general conclusion, the 1-naphthaloyl capping group (1-Nap) is more efficacious in triggering gelation of dehydrodipeptides than the 2-naphthylacethyl group (2-Naph). This result reinstates the 1-Nap group, generally overlooked, as a capping group worth exploring further in the design of novel supramolecular hydrogels.

### 2.3. Self-Assembly Studies

#### 2.3.1. Fluorescence and Circular Dichroism (CD) Spectroscopy

**Figure 3:**
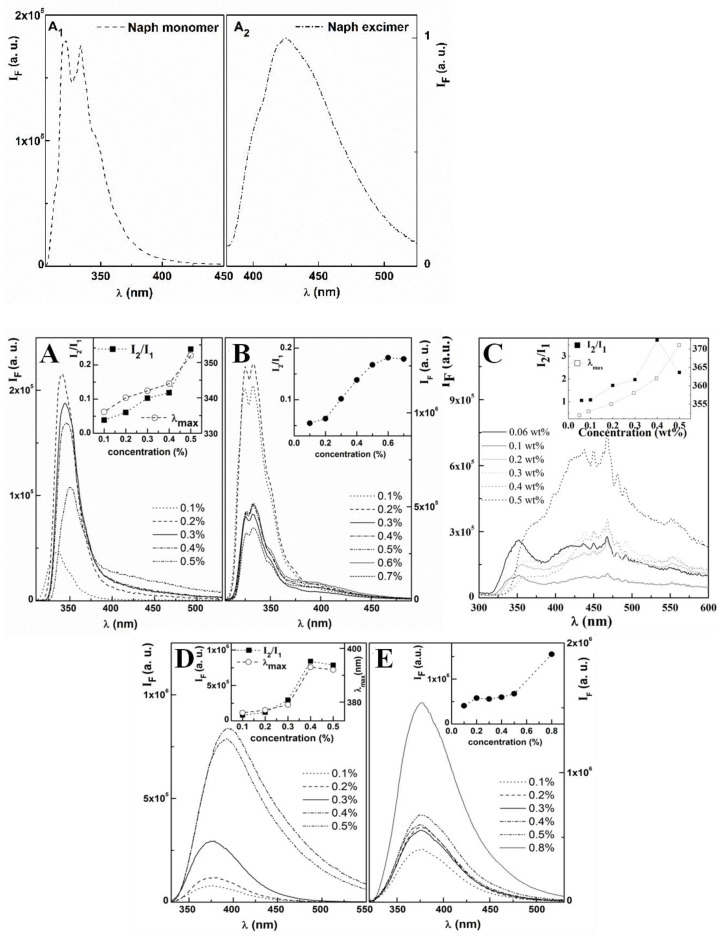
Fluorescence spectra (λ_exc_ = 280 nm) of naphthalene in ethanol: (**A_1_**) monomeric, non-associated form, at 10^−5^ M; (**A_2_**) associated form, excimer, at 10^−1^ M. (**A**–**E**): Concentration dependence of the fluorescence emission (λ_exc_ = 280 nm) of hydrogelators/hydrogels: (**A**) **1a** (phosphate buffer, 0.1 M, pH 8); (**B**) **1b** (NaOH/GDL, pH 6); (**C**) **1c** (NaOH/GDL, pH 4); (**D**) **2a** (NaOH/GDL, pH 6); (**E**) **2b** (NaOH/GDL, pH 6). Insets: concentration dependence of the wavelength of maximum emission for the non-aggregated form, λ_max_, and intensity ratio I_2_/I_1_. For hydrogelators/hydrogels **1c** (**C**) and **2b** (**E**), the wavelength of maximum emission does not show concentration dependence.

Both hydrogelators and hydrogels **1a**–**c** exhibit fluorescence emission bands at wavelengths around 350 nm characteristic of non-conjugated substituted naphthalene compounds (Figure 3A_1_,A_2_) [32].

Molecular self-assembly was studied by monitoring the intrinsic fluorescence (λ_exc_ = 280 nm) of the naphthalene-derived capping group (2-Naph or 1-Nap), as the phenylalanine and dehydrophenylalanine amino acid residues are not excited at 280 nm [23,24,25]. For hydrogelators and hydrogels **1a**–**c**, the sharp intense emission band around 340 nm is attributed to the emission of the non-aggregated (monomeric) (I_1_) form. For hydrogels **2a** and **2b** N-capped with the 1-Nap group, the I_1_ emission band, at around 380 nm, is broad and non-structured, provably due to conjugation [33]. A second broad band (I_2_) with a wavelength of maximum emission near 400 nm for compound **1c**, and in the range of 400–450 nm for hydrogelators **1a** and **1b**, is assigned to emissive aggregates formed by self-assembly of hydrogelator molecules by intermolecular π–π stacking interactions [23,24,25]. The aggregate band (I_2_) is not apparent in the fluorescence spectra of the hydrogelator/hydrogel **2b** (Figure 3). The concentration dependence of the wavelength of maximum emission for the non-aggregated form (I_1_) and the intensity ratio (I_2_/I_1_) was studied in a concentration range encompassing the CGC values determined empirically (Table 1, Figure 3). The concentration-dependent red shift of the wavelength of maximum emission for the non-aggregated form of compounds **1a**, **1b** and **2a**, is accompanied by an enhancement of the I_2_/I_1_ intensity ratio, indicating that the aggregation (self-assembly) is driven by π–π stacking interactions [23,24,25]. The wavelength of maximum emission for compounds **1c** and **2b** does not exhibit concentration dependence, suggesting less effective aggregation (self-assembly) through intermolecular π–π stacking interactions.

Hydrogelators **1a**, **1b** and **1c** display similar absorbance spectral signatures characteristic of naphthalene and substituted naphthalenes [34]: the medium intensity broad band between 250 and 300 nm (λ_max_ 277 nm) and the intense band around 210–230 nm (λ_max_ 224 nm) are assigned to naphthalene-centered π–π* long-axis and short-axis polarized transitions, respectively. The peptide bond n-π* and π–π* transitions originate an intense band in the range 180–200 nm (λ_max_ 190 nm). The absorption band between 260–290 nm appears, for hydrogelator **1c**, as a shoulder of the band at 225 nm. Despite considerable shifts compared to compound **1a**, compounds **2a** and **2b** still display spectral features characteristic of naphthalene: whilst the band at λ_max_ = 280 nm is red-shifted, the bands at lower wavelengths are blue-shifted (Supplementary Materials Figure S4).

**Figure 4:**
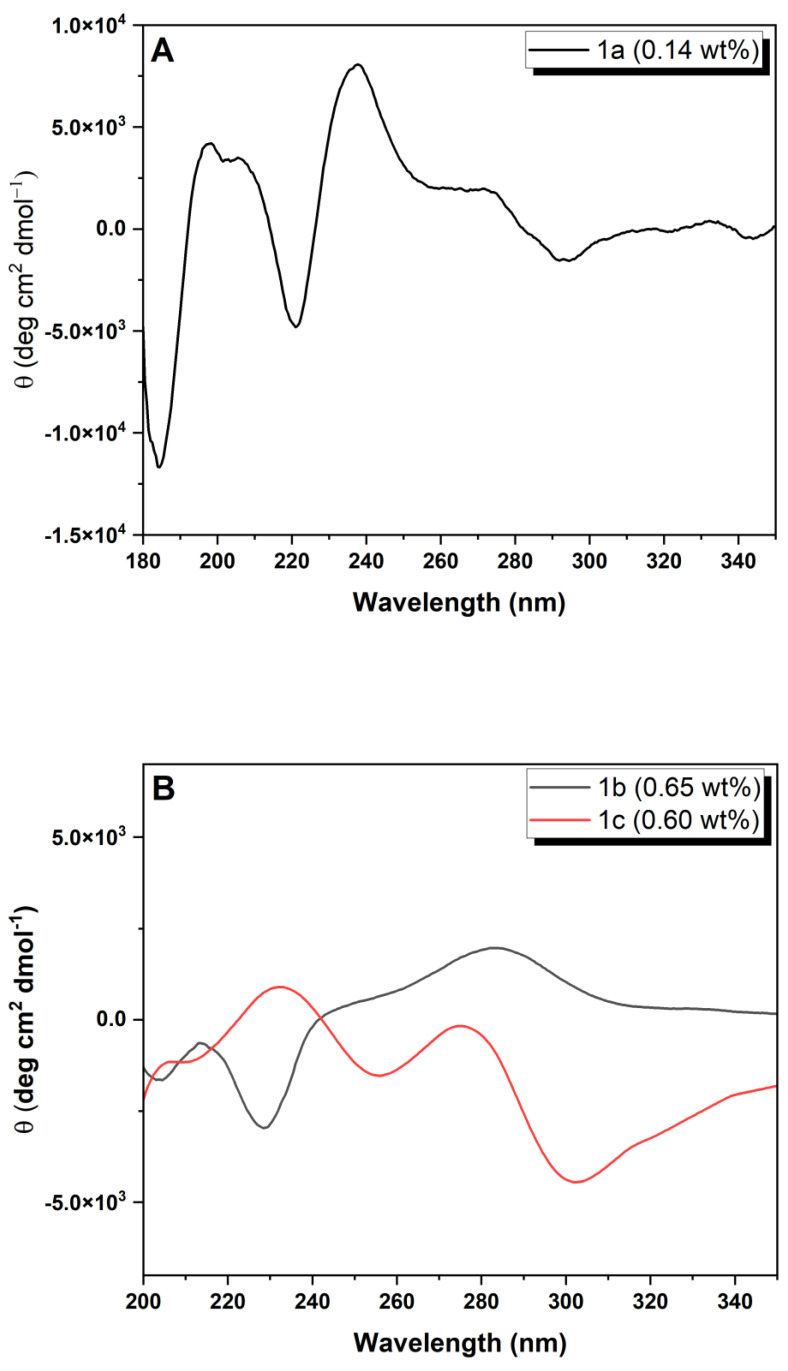

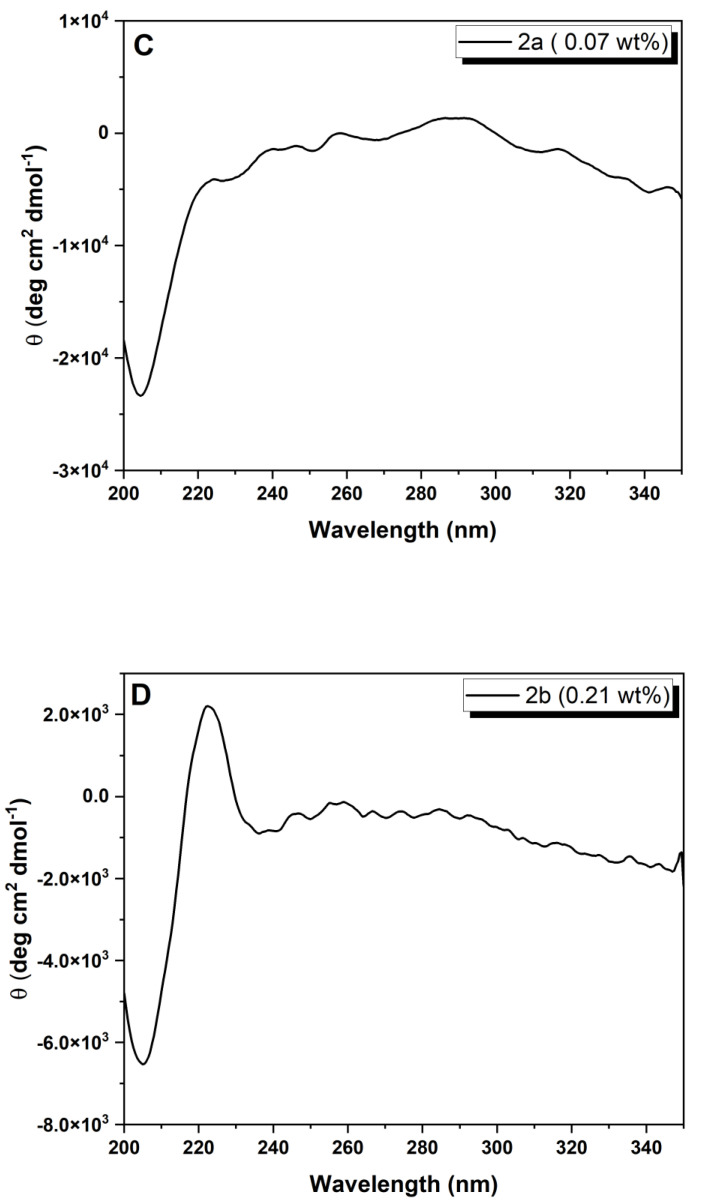
CD spectra of peptides: (**A**) **1a** (0.14 wt%, phosphate buffer 0.1 M, pH 8.0); (**B**) **1b** (0.65 wt%, pH 6) and **1c** (0.60 wt%, pH 4); (**C**) **2a** (0.07 wt%, pH 5); (**D**) **2b** (0.21 wt%, pH 5).

CD spectra were acquired for peptides **1a**–**c** and **2a** and **2b** in the gel phase, at concentrations of the order of magnitude of the CGC (Table 1) to get insights into peptide self-assembly (Figure 4).

**Figure 5:**
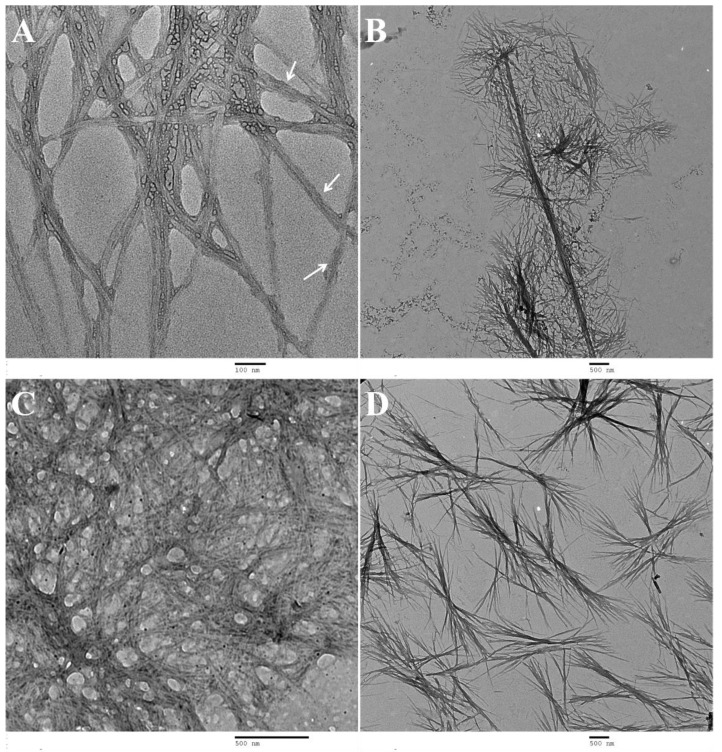
TEM images: (**A**) **1a** (0.028 wt%, pH 8), scale 100 nm (arrows indicate the twisting seen in the fibres); (**B**) **1b** (0.65 wt%, pH 6), scale 500 nm; (**C**) **2a** (0.014 wt%, pH 6), scale 500 nm; (**D**) **2b** (0.04 wt%, pH 6), scale 500 nm.

The CD spectrum of compound **1a** at the CGC (0.14 wt%, phosphate buffer pH 8.0) exhibits features characteristic of a twisted β-sheet secondary structure: negative and positive peaks at 184 nm and 198 nm, respectively, assigned to exciton couplets of the π–π* transition of the peptide backbone. The shoulder at 211 nm and the negative and positive Cotton effects at 221 nm and 238 nm can be assigned to overlap of the n−π* transition of the peptide backbone and the naphthalene absorption at 224 nm (Supplementary Materials; Figure S4) [35,36]. The Cotton effect at 224 nm indicates chiral stacking of the naphthalene aromatic rings. The positive shoulder at 275 nm and the weak broad negative band at 294 nm, assigned to the naphthalene π−π* short-axis polarized transitions, indicate intermolecular interactions in the hydrogel fibers [27,28,29]. The chirality of the twisted β-sheet arrangement translates into hydrogel **1a** fibers showing helicity, seen in the TEM image of gel **1a** (Figure 5A) [37,38]. CD spectra of hydrogels obtained from compounds **1b**,**c** and **2a**,**b** were recorded in the wavelength range 200–350 nm. The hydrolysis product of GDL, *D*-gluconic acid, is CD-active, originating a strong negative band in CD bellow 200 nm, which can obscure the CD peptide band originating from the π−π* UV transition (180–200 nm) [28]. Moreover, overlap of the peptide n−π* transition (UV absorption at 210–230 nm) and the Cotton effect of the naphthalene moiety (UV absorption around 225 nm), makes the determination of the secondary structure of the peptide backbone ambiguous for peptides **1b**,**c** and **2a**,**b**. The CD spectrum of hydrogel **1b** (0.65 wt%, pH 6) (Figure 4B) is similar to that of hydrogel **1a** (Figure 4A), with a negative band at around 230 nm, corresponding possibly to the Cotton effect observed for compound **1a** at 224 nm and a broad positive band around 280 nm. The CD spectrum of hydrogel **1c** (0.60 wt%) (Figure 4B) exhibits positive Cotton effects at 232 and 275 nm and a broad negative band at 302 nm. Hydrogels **1b** and **1c** seem to display opposite chiral stacking arrangements of the naphthalene aromatic rings in the self-assembled nanofibers, as revealed by the opposite sign of the Cotton effects at around 225 nm [39]. CD spectra of hydrogels **2a** and **2b** present some similarities with the CD spectrum of hydrogel **1a:** strong positive Cotton effects at around 220 nm and a negative band at 204 nm, indicative of chiral stacking of the naphthalene aromatic rings. The CD results highlight the determining contribution of naphthalene π–π stacking interactions to the self-assembly.

#### 2.3.2. STEM and TEM Imaging

Transmission Electron Microscopy (TEM) and Scanning Transmission Electron Microscopy (STEM) were used to analyze the nanostructures formed by hydrogels **1a**,**b** and **2a**,**b** (Figure 5) (Supplementary Materials Figures S5 and S6). A fibrillar network is apparent for all hydrogels, despite difficulties in obtaining good-quality images for gel **1c**. The fibrillar nature of hydrogel **1c** is confirmed by STEM images of the gel (Supplementary Materials Figure S5). TEM images of peptides **1a** and **2a** (Figure 5A,D, respectively) display a heterogeneous fibrillar structure, with variable density and extent of cross-linking. The diameter of the fibers is in the range 11–38 nm and 13–23 nm, for compounds **1a** and **2a**, respectively. STEM analysis of peptide **1a** as gel (0.5 wt%, pH 6) afforded a picture similar to the TEM analysis. Interestingly, twisting could be identified in some fibers of peptide **1a** in the TEM images, in accordance with the CD results which indicate that peptide **1a** self-assembles into twisted β-sheet secondary structures [37,38,39]. Peptides **1b** and **2b** exhibit bundles of fibers in solution (Figure 5B,D). The fibers within the bundles show no specific orientation for peptide **1b** but are straight and long (3–4 μm in length) for hydrogel **2b** and do not seem to bend and intertwine. The bundles of fibers of peptide **1b** are connected by thinner fibers with thicknesses between 14 and 17 nm. For peptides **1b** and **2b**, the fibers seem not to have uniform thickness throughout their length, ranging from 18–30 nm for peptide **1b** and 19–50 nm for peptide **2b**, suggesting some degree of twisting [37,38,39].

Hydrogelators **1a** and **2a**, the same peptide with different capping groups, display similar nanofiber size and morphology and analogous gel networks. This suggests that the peptide structure, instead of the capping group, determines the shape and size of the gel fibers and the 3D network architecture [39].

#### 2.3.3. Rheology

**Figure 6:**
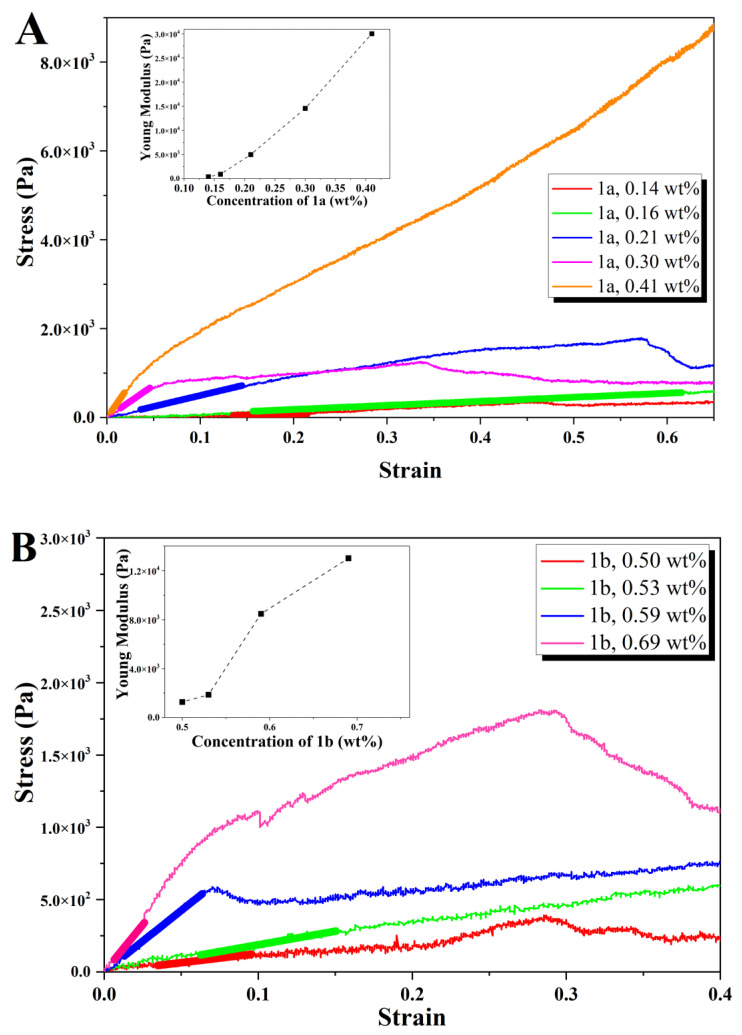
Stress-strain response curves for penetration tests for hydrogels at 25 μm·s^−1^ plunger speed: (**A**) hydrogel **1a**; inset: concentration dependence of Young’s modulus. (**B**) hydrogel **1b**; inset: concentration dependence of Young’s modulus. Young’s modulus was determined from the slope of the linear regime of the stress–strain response curves (indicated by bold lines).

**Figure 7:**
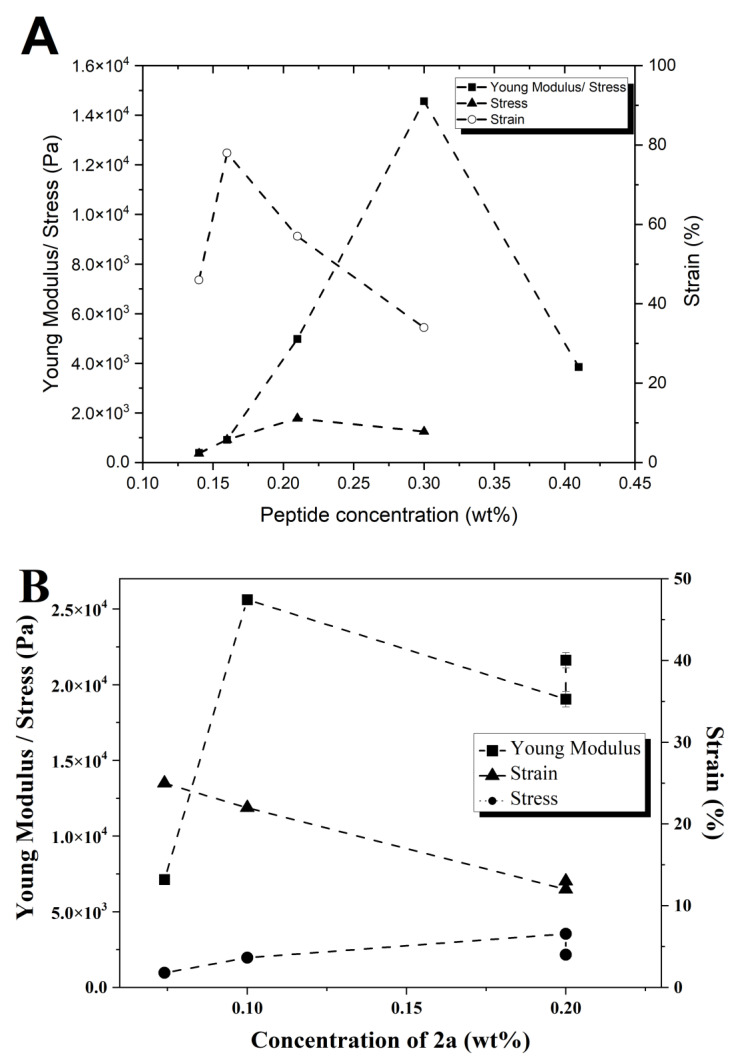

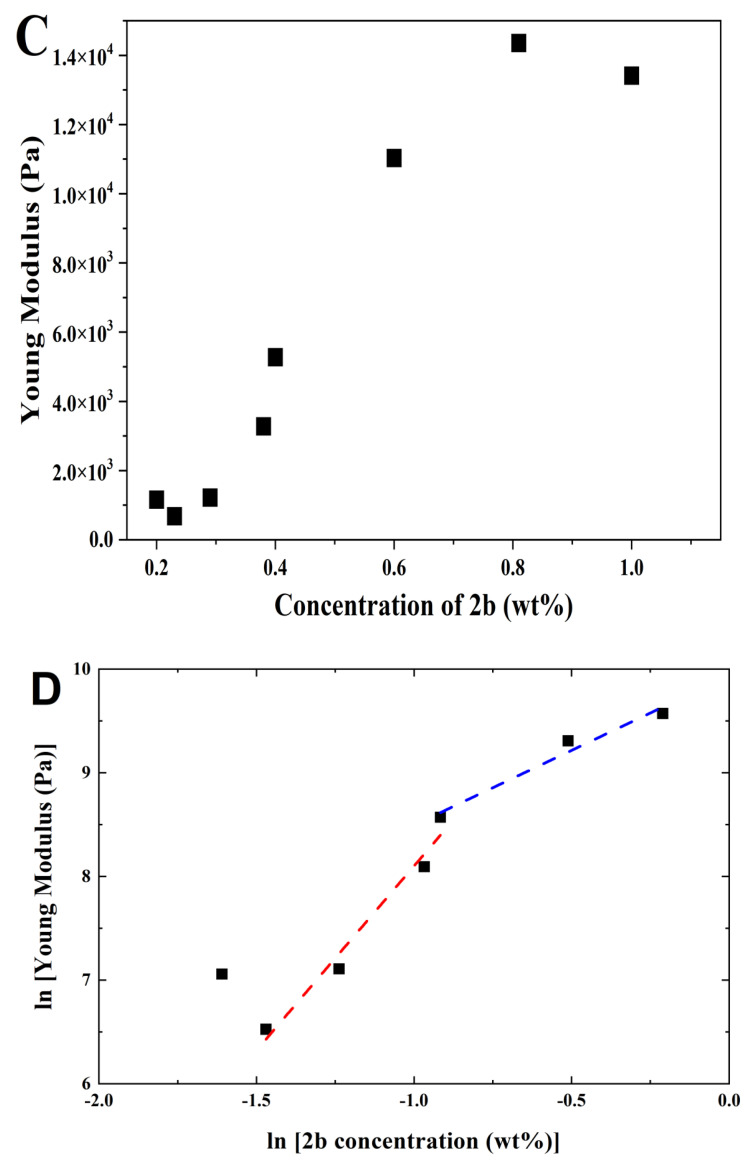
Concentration dependence of Young’s modulus and stress and strain at break for: (**A**) gel **1a**; (**B**) gel **2a**. (**C**,**D**) Concentration dependence of the elasticity for gel **2b**. The red and blue lines represent the linear fitting [blue line: ln(E) = 11.66 ± 0.51 + ln(conc) 3.56 ± 0.44, r^2^ = 0.96; red line: ln(E) = 9.94 ± 0.16 + ln(conc) 1.44 ± 0.26, r^2^ = 0.94].

Penetration tests, performed on (pre)gelled samples, were used to determine the elasticity of the gels of peptides **1a**,**b** and **2a**,**b**, following a protocol described previously [28]. This study focuses on the viscoelastic comparison between gels and not on the gelation properties of the gels. The slow gelation kinetics of dehydropeptide-based hydrogels limits the amount of data that can be acquired by dynamic shear rheology, performed on samples samples gelling in the rheometer over a 24 h period [18,19,20]. Gels of peptide **1c** revealed unsuitable, too weak, for characterization by the penetration tests protocol applied to gels **1a**,**b** and **2a**,**b**. The concentration dependence of Young’s modulus (E) was obtained for hydrogels **1a**,**b** and **2a**,**b** (Figure 6 and Figure 7) (Supplementary Material, Figure S7). Young’s modulus E was converted to the shear modulus G, which is of the same order of magnitude of the storage modulus—G′, usually determined by rotational rheometry as the parameter characterizing the elasticity of supramolecular hydrogels. Assuming that gels are incompressible, G′ ≈ G = E/3 (Table 2) [40].

For hydrogels **1a** (0.14–0.40 wt%, phosphate buffer pH 8) and **2a** (0.074–0.20 wt%, GDL, pH 6) Young’s modulus increases with peptide concentration until a maximum in elasticity is reached, at 0.30 wt% and 0.10 wt% for hydrogels **1a** and **2a**, respectively (Figure 7A,B). Regarding the concentration dependence of the stress and strain at which gels break, the maximum breaking stress is attained for hydrogel **1a** at 0.2 wt%, with one of the highest yielding strengths. Therefore, gel **1a** is hard to break when compared to other gels since larger strains need to be applied. However, in contrast to other gels, the rheological data for hydrogel **1a** were measured after thermosetting, which may explain the larger strain at break [41]. For hydrogel **2a**, the concentration dependence of the stress and strain at break show that the strain decreases linearly and the stress shows low concentration dependence. The similar elasticity trend observed for hydrogels **1a** and **2a**, sharing the Phe-ΔPhe peptide sequence capped with the 2-Naph and 1-Nap groups, respectively, is explained by their comparable fibrillar networks seen in the TEM and STEM experiments (Figure 5A,D). The elasticity maxima observed for both hydrogels suggest that a maximum density of the fibrillar network is reached, after which the gel elasticity decreases [42]. Compound **2a** formed stronger gels than **1a** (G′ 8.5 and 4.9 kPa, for hydrogels **2a** and **1a**, respectively), which is in agreement with the higher density of the fiber network seen in the TEM/STEM images of hydrogel **2a**. The elasticity concentration dependence for gels **1b** (0.50–0.69 wt%) and **2b** (0.20–1.0 wt%) shows a similar trend, different from that seen for hydrogels **1a** and **2a**: non-linear increase with peptide concentration stabilizing at around 0.69 wt% and 0.8 wt%, for hydrogelators **1b** and **2b**, respectively. On closer inspection, the concentration dependence of the elasticity of gel **2b** shows two general trends governed by power laws with exponents 3.5 ± 0.5 and 1.4 ± 0.3 for hydrogelator concentrations below and above 0.6 wt%, respectively, reflecting probably the fractal dimensions of the fibrils (Figure 7C) [42]. The TEM/STEM images of peptides **1b** and **2b** reveal bundles of fibers in solution (Figure 5B,E). At low hydrogelator concentrations, some fibers in the bundles are not connected to other bundles, acting as free ends, not mechanically effective. At higher concentrations, all fibrils contribute to gel elasticity. Thus, the concentration scaling (exponent 1.5) expected for enthalpic (rigid) junctions made by bundles of fibers is attained. Similar concentration regimes for the elastic behavior was found for gels of synthetic polymers and polysaccharides [43].

#### 2.3.4. Computer Modelling

**Figure 8:**
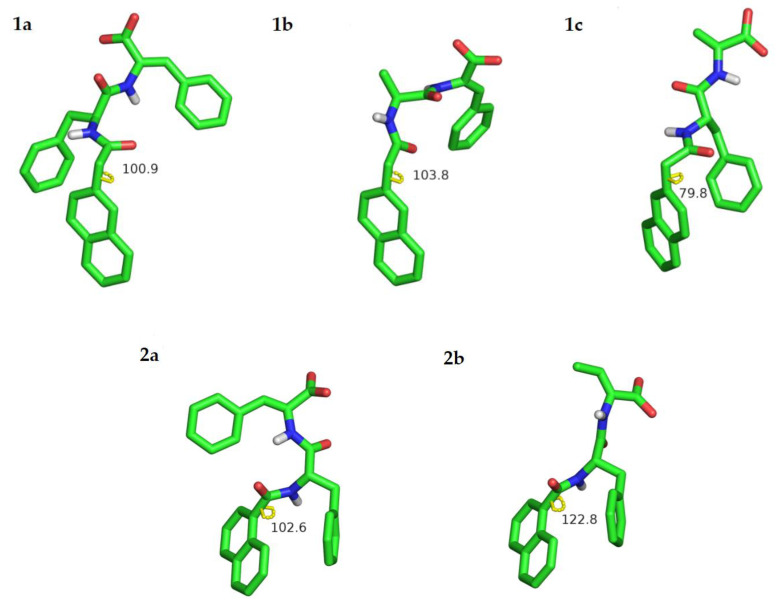
Most populated conformations obtained for hydrogelators **1a**–**c** and **2a** and **2b** in water from MD simulations refined at the BLYP-D3/DEF2-TZVP level. Only polar hydrogen atoms are shown. C atoms are represented in green, H in gray, O in red and N in blue. The yellow lines and the associated figures represent the dihedral angle describing the orientation of the naphthalene ring in relation to the amide plane.

The 2-Naph protecting group is widely deployed for gelation of low-molecular-weight peptides [34,36]. As far as we are aware, the 1-Nap group was mostly disregarded as a capping group for the synthesis of low-molecular-weight peptide gelators [33]. In this work, we demonstrate that 1-Nap-protected hydrogelators display higher self-assembly and gelation propensity than 2-Naph-capped hydrogelators. These results seem counterintuitive from the chemical point of view. In 1-Nap-protected peptides, the naphthalene ring and the peptide backbone plane are likely to adopt a near perpendicular conformation, dihedral angle around 90°, which presumably precludes intermolecular aromatic π–π stacking interactions and hydrogen bonding [33]. Classical molecular dynamics (MD) simulations were carried out to get insights into structural effects of the 2-Naph and 1-Nap capping group on the self-assembly of the dehydrodipeptides (see methods for details). Conformational populations and intra- and inter-molecular interactions were explicitly computed for hydrogelators **1a**–**c** and **2a** and **2b**. First, single solvated dehydrodipeptides were modeled with MD for 100 ns at infinite dilution conditions. The trajectories were analyzed and the most likely conformations were extracted and refined with a DFT Hamiltonian augmented by an implicit water model. Dehydropeptides were found to populate mostly a single conformation for more than 92% of the simulation time (Figure 8).

**Figure 9:**
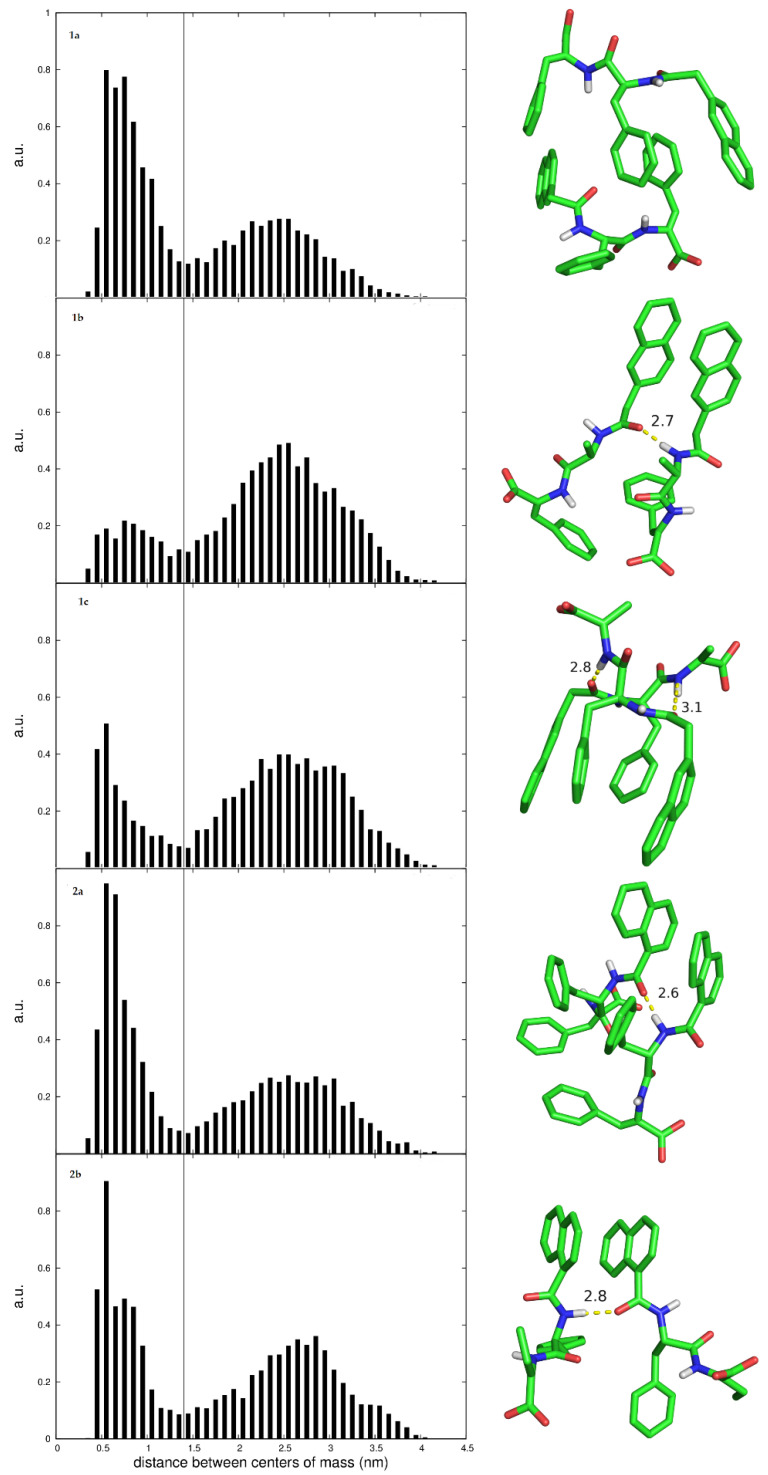
Radial distribution function of the centers of mass for two peptide molecules, together with illustrative snapshots of the molecular structure of bound dimers. Only polar hydrogen atoms are shown. C atoms are represented in green, H in gray, O in red and N in blue. Hydrogen bonds are represented by yellow dashed lines and the associated numbers represent the length of the hydrogen bonds.

The dihedral angle describing the orientation of the naphthalene ring of the protecting group in relation to the amide plane was used to characterize the most stable conformation of the dehydropeptides (Figure 8). In all cases, the naphthalene ring was found to be approximately perpendicular to the amide plane for both the 2-Naph and 1-Nap protected hydrogelators. In addition, the aggregation kinetics of two hydrogelator molecules, i.e., dimer formation, was also studied with MD simulations. For this, the distance between the centers of mass of the two peptide molecules was monitored during the 60 ns production window. The intermolecular radial distribution function was used as an aggregation metric. Peptides were considered bound when their centers of mass sit at distances closer than the cutoff intermolecular distance of 1.4 nm (Figure 9).

Figure 9 shows snapshots of the molecular structure of dimer dehydropeptides in water—the peptide centers of mass are at typical 0.4–0.7 nm distances, illustrating the intra and intermolecular π–π stacking interactions and hydrogen bonding, which collectively contribute to molecular self-assembly. For contact distances, the area under the distribution curve represents the fraction of bound dimers through the simulation. A larger and sharper population can be correlated with a higher propensity for self-assembly. Accordingly, peptides **1a**, **2a** and **2b** are predicted to have a higher aggregation propensity than peptides **1b** and **1c**. These results agree qualitatively with the CGC values determined experimentally (Table 1). Moreover, dimer **2a**, displaying the largest and sharpest bound population, has also the lowest experimental CGC, corroborating the notion that the 1-Nap protecting group is a better gelation inducer than the 2-Naph group. A snapshot of the bound conformations for dimer **2a** shows a parallel arrangement of the peptide molecules, establishing an extended array of intermolecular π–π stacking interactions between the naphthalene groups of the 1-Nap protecting group and the Phe and ΔPhe residues, together with hydrogen bonding between the N-terminal amide groups. Peptide **1a** dimers display an antiparallel (head-to-tail) arrangement, which allows for an extended array of sandwich-type π–π stacking interactions, involving simultaneously the Phe, ΔPhe and the naphthalene moiety of the 2-Naph protecting group, but precludes hydrogen bonding of the peptide scaffold. Hydrogen bonding allied to direct π–π stacking interactions of the naphthalene groups suggests more favorable intermolecular interactions for dimers of **2a** in comparison to **1a**, and consequently higher self-assembly propensity, in agreement with the experimental observations.

## 3. Conclusions

In this work, we found that dehydrodipeptides 1-Nap-L-Phe-Z-ΔPhe-OH and 1-Nap-L-Phe-Z-ΔAbu-OH, N-capped with the 1-naphthoyl (1-Nap) group, are very efficacious hydrogelators (CGC = 0.07 and 0.2 wt%, respectively, GDL, pH 5). Moreover, peptide 1-Nap-L-Phe-Z-ΔPhe-OH is a more efficacious gelator than the same peptide capped with the 2-naphthylacetyl (2-Naph) group—2-Naph-L-Phe-Z-ΔPhe-OH (CGC = 0.2 and 0.5 wt%, respectively, GDL, pH 6.0). The gelation-enhancing effect of the 1-Nap capping group compared to 2-Naph is further supported by comparison with the hydrogelators Npx-L-Phe-Z-ΔPhe-OH and Npx-L-Phe-Z-ΔAbu-OH (CGC = 0.4 wt% PBS buffer pH 8.0 and pH 6, respectively) N-capped with the NSAID naphthalene-derived drug naproxen [29]. TEM/STEM microscopy suggests that the size of the hydrogel fibers and the architecture of the gel network, cross-linked networks versus cross-linked fiber bundles, are determined by the peptide sequence instead of the structure of the capping group. Hydrogel elasticity concentration dependence was explained by rheological models based on cross-linked gel fiber networks and cross-linked fiber bundles, as seen in the microscopy study. Fluorescence and CD spectroscopy studies point to intermolecular π–π stacking interactions of the naphthalene moieties of the protecting groups as the self-assembly driving force. MD simulations revealed intermolecular naphthalene-naphthalene π–π stacking interactions and hydrogen bonding for the 1-Nap-protected peptides only, thus predicting a higher self-assembly propensity for the 1-Nap-protected hydrogelators. The MD simulation results are in good agreement with the gelation studies and the fluorescence and CD results. For the dehydrodipeptide ensemble studied, the 1-Nap group is more efficacious in promoting peptide self-assembly and gelation than the 2-Naph group. The 1-Nap group was essentially overlooked by researchers as a capping group for low-molecular-weight peptide-based hydrogels [33]. This study adds the 1-Nap group to the palette of capping groups available for the preparation of efficacious low-molecular-weight peptide-based hydrogels.

## 4. Materials and Methods

The synthetic procedures and compound characterization is included in the PhD thesis of Dr. Helena Vilaça, accessible in the Repositorium of the University of Minho (RepositoriUM; http://hdl.handle.net/1822/40563; accessed on 10 March 2023) [44]. Melting points (mp, °C) were determined in a Gallenkamp apparatus, Cambridge, UK and are uncorrected. ^1^H and ^13^C NMR spectra were recorded on a Bruker Avance III (Billerica, MA, USA) at 400 and 100.6 MHz, respectively, or in a Varian Unity Plus 300 (Hansen Way Palo Alto, CA, USA) at 300 and 75.4 MHz, respectively. 1H-1H spin-spin decoupling and DEPT θ 45° were used. HMQC and HMBC were used to attribute some signals. Chemical shifts (δ) are given in parts per million (ppm), downfield from tetramethylsilane (TMS), and coupling constants (J) in Hertz (Hz). High-resolution mass spectrometry (HRMS) data were recorded by the mass spectrometry service of the University of Vigo, Spain. Elemental analysis was performed on a LECO CHNS 932 (Lakeview, IL, USA) elemental analyzer. Column chromatography was performed on Macherey–Nagel silica gel 230–400 mesh. Petroleum ether refers to the boiling range 40–60 °C. Some reactions were monitored by thin layer chromatography (TLC), using pre-coated TLC-sheets Alugram Xtra SIL G/UV254. The sheets were revealed in UV light (254 nm). Acetonitrile (ACN) was dried over silica and calcium hydride (CaH_2_) and then distilled and stored under molecular sieves.

Synthesis: Compounds **3a** (CAS 7524-50-7, Fluorochem cat. nº 003826, UK) and **3b** (CAS 2491-20-5, Fluorochem cat. nº M02946, UK) are commercially available. The synthesis of compounds **9a** and **9b** was described elsewhere [28,29].

Synthesis of amino acid methyl esters (**3a**,**b**): To methanol (1 M of amino acid) in an ice bath was slowly added thionyl chloride (3.4 equiv.). The amino acid was added slowly and the mixture was left stirring at 40 °C for 4 h. The solvent was removed under reduced pressure and ethyl ether was added. The mixture was stored in the freezer for 1 h and then the solid was filtered.

H-L-Phe-OMe, HCl (**3a**): H-L-Phe-OH (5.26 g, 31.8 mmol) gave compound **3a** (6.72 g, 98%) as a white solid; mp: 153–155 °C (mplit.: 156.0–160.0 °C); ^1^H NMR (400 MHz, DMSO-*d_6_*, δ): 3.09 (dd, *J* = 7.4 and 14.0 Hz, 1H, βCH), 3.21 (dd, *J* = 5.6 and 14.0 Hz, 1H, βCH), 3.64 (s, 3H, OCH_3_), 4.21 (dd, *J* = 5.6 and 7.4 Hz, 1H, αCH), 7.22–7.34 (m, 5H, Ar H), 8.77 (s, 3H, NH_3_^+^).

H-L-Ala-OMe,HCl (**3b**): H-L-Ala-OH (5.18 g, 58.1 mmol) gave compound **3b** (8.11 g, quant.) as a white solid; ^1^H NMR (400 MHz, DMSO-*d_6_*, δ): 1.41 (d, *J* = 6.8 Hz, 3H, CH_3_), 3.71 (s, 3H, OCH_3_), 4.11 (brs, 1H, αCH), 8.73 (brs, 3H, NH_3_^+^).

Synthesis of 2-naphthylacetylamino acids (**4a**,**b**): 2-Naphthylacetic acid was dissolved in acetonitrile (10 mL mmol-1) and put in an ice bath. HOBt (1.0 equiv.), DCC (1.0 equiv.), amino acid methyl ester (1.0 equiv.) and triethylamine (2.0 equiv.) were added, waiting about 2 min between each addition. The mixture was left stirring at rt overnight (~18 h). The urea was filtered and the solvent removed under reduced pressure. Acetone was added and the mixture was stored in the freezer for 2 h. The urea was filtered again. Evaporation at reduced pressure gave a residue that was partitioned between ethyl acetate (50 mL) and KHSO_4_ (30 mL, 1 M). The organic phase was thoroughly washed with _KHSO4_ (1 M), NaHCO_3_ (1 M) and brine (3 × 30 mL, each), and dried with MgSO_4_. Removal of the solvent afforded compounds **4a**,**b**.

2-Naph-L-Phe-OMe (**4a**): 2-Naphthylacetic acid (0.37 g, 2.00 mmol) and H-L-Phe-OMe,HCl (**3a**) gave compound **4a** as a white solid (0.64 g, 93%); mp: 85.0–87.0 °C; ^1^H NMR (400 MHz, CDCl_3_, δ): 3.01 (dq, *J* = 5.6 and 14.0 Hz, 2H, βCH_2_), 3.70 (s, 3H, OCH_3_), 3.72 (s, 2H, CH_2_), 4.86 (td, *J* = 5.6 and 7.6 Hz, 1H, αCH), 5.83 (d, *J* = 7.2 Hz, 1H, NH), 6.79 (d, *J* = 7.4 Hz, 2H, H_o_ Phe), 6.99 (t, *J* = 8.0 Hz, 2H, H_m_ Phe), 7.10 (tt, *J* = 2.0 and 7.4 Hz, 1H, H_p_ Phe), 7.30 (dd, *J* = 1.6 and 8.4 Hz, 1H, H-3 Naph), 7.49–7.54 (m, 2H, 2 × Ar H Naph), 7.66 (s, 1H, H-1 Naph), 7.79–7.87 (m, 3H, 3 × Ar H Naph); ^13^C NMR (100.6 MHz, CDCl_3_, δ): 37.52 (βCH_2_), 43.81 (CH_2_), 52.30 (OCH_3_), 52.89 (αCH), 126.05 (CH Naph), 126.37 (CH Naph), 126.97 (C_p_ Phe), 127.19 (CH-3 Naph), 127.70 (2 × CH Naph), 128.26 (CH-1 Naph), 128.40 (C_m_ Phe), 128.80 (CH Naph), 128.99 (C_o_ Phe), 131.85 (C-2 Naph), 132.57 (C-4a Naph), 133.56 (C-8a Naph), 135.33 (C_i_ Phe), 170.42 (C=O Naph), 171.72 (C=O Phe); HRMS (ESI) *m*/*z*: [M+H]+ calcd for C_22_H_22_NO_3_^+^ 348.1594; found, 348.1592.

2-Naph-L-Ala-OMe (**4b**): 2-Naphthylacetic acid (0.55 g, 2.95 mmol) and H-L-Ala-OMe,HCl (**3b**) gave compound **4b** as a white solid (0.62 g, 78%); mp: 93.5–96.0 °C; ^1^H NMR (400 MHz, CDCl_3_, δ): 1.33 (d, *J* = 7.2 Hz, 3H, βCH_3_), 3.71 (s, 3H, OCH_3_), 3.77 (s, 2H, CH_2_), 4.61 (quint, *J* = 7.2 Hz, 1H, αCH), 6.05 (d, *J* = 6.4 Hz, 1H, NH), 7.41 (dd, *J* = 2.0 and 8.4 Hz, 1H, H-3), 7.46–7.53 (m, 2H, Ar H), 7.75 (s, 1H, H-1), 7.82–7.87 (m, 3H, Ar H); ^13^C NMR (100.6 MHz, CDCl_3_, δ): 18.26 (βCH_3_), 43.72 (CH_2_), 48.10 (αCH), 52.40 (OCH_3_), 125.99 (CH), 126.35 (CH), 127.19 (CH-3), 127.66 (CH), 127.70 (CH), 128.23 (CH-1), 128.74 (CH), 131.96 (C-2), 132.54 (C-4a), 133.54 (C-8a), 170.41 (C=O Naph), 173.30 (C=O Ala); HRMS (ESI) *m*/*z*: [M+H]^+^ calcd for C_16_H_18_NO_3_^+^ 272.1281; found, 272.1280.

Synthesis of compounds **5a**,**b**: The amino acid derivative was dissolved in methanol (until 10 mL) and NaOH (1 M) (1.5 equiv.). The reaction was followed by TLC (Thin Layer Chromatography) until no starting material was detected. The organic solvent was removed under reduced pressure, and the reaction mixture was acidified to pH 3 with KHSO4 (1 M). The solid formed was filtered, affording compounds **5a**,**b**.

2-Naph-L-Phe-OH (**5a**): 2-Naph-L-Phe-OMe (**4a**) (0.55 g, 1.58 mmol) gave compound **5a** as a white solid (0.48 g, 91%); mp: 145.0–147.0 °C; ^1^H NMR (400 MHz, DMSO-*d_6_*, δ): 2.88 (dd, *J* = 9.6 and 14.0 Hz, 1H, βCH), 3.07 (dd, *J* = 4.8 and 14.0 Hz, 1H, βCH), 3.58 (dd, *J* = 14.0 and 21.2 Hz, 2H, CH_2_), 4.41–4.48 (m, 1H, αCH), 7.19 (brs, 5H, Ar H Phe), 7.27 (dd, *J* = 1.6 and 8.4 Hz, 1H, Ar H), 7.43–7.50 (m, 2H, Ar H), 7.65 (s, 1H, Ar H), 7.76–7.80 (m, 2H, Ar H), 7.85 (d, *J* = 8.0 Hz, 1H, Ar H), 8.44 (d, *J* = 8.0 Hz, 1H, NH), 12.63 (brs, 1H, CO_2_H); ^13^C NMR (100.6 MHz, DMSO-*d_6_*, δ): 36.71 (βCH_2_), 42.09 (CH_2_), 53.49 (αCH), 125.45 (CH), 125.99 (CH), 126.36 (C_p_ Phe), 127.21 (CH), 127.32 (CH), 127.43 (CH), 127.45 (CH), 127.56 (CH), 128.10 (C_m_ Phe), 129.11 (C_o_ Phe), 131.72 (C), 132.91 (C), 133.85 (C), 137.53 (C_i_ Phe), 169.96 (C=O), 172.99 (CO_2_H); HRMS (ESI) *m*/*z*: [M+H]^+^ calcd for C_21_H_20_NO_3_^+^ 334.1438; found, 334.1435.

2-Naph-L-Ala-OH (**5b**): 2-Naph-L-Ala-OMe (**4b**) (0.37 g, 1.36 mmol) gave compound **5b** as a white solid (0.28 g, 80%); mp: decomposes above 157.0 °C; ^1^H NMR (400 MHz, DMSO-*d_6_*, δ): 1.27 (d, *J* = 7.2 Hz, 3H, βCH_3_), 3.62 (s, 2H, CH_2_), 4.21 (t, *J* = 7.2 Hz, 1H, αCH), 7.41–7.48 (m, 3H, Ar H), 7.76 (s, 1H, Ar H), 7.82–7.87 (m, 3H, Ar H), 8.45 (d, *J* = 7.2 Hz, 1H, NH), 12.49 (brs, 1H, CO_2_H); ^13^C NMR (100.6 MHz, DMSO-*d_6_*, δ): 17.21 (βCH_3_), 41.94 (CH_2_), 47.58 (αCH), 125.46 (CH), 126.02 (CH), 127.23 (CH), 127.34 (CH), 127.43 (CH), 127.48 (CH), 127.63 (CH), 131.73 (C), 132.94 (C), 133.94 (C), 169.83 (C=O), 174.13 (CO_2_H); HRMS (ESI) *m*/*z*: [M+H]^+^ calcd for C_15_H_16_NO_3_^+^ 258.1125; found, 258.1125.

Synthesis of dipeptide derivatives (**6a**–**c**): The procedure is similar to the one described for amino acid derivatives **4a**,**b**, but 2-naphthylacetic acid is substituted by the N-protected amino acid derivative (**5a**,**b**).

2-Naph-L-Phe-D,L-Phe(β-OH)-OMe (**6a**): 2-Naph-L-Phe-OH (**5a**) (0.24 g, 0.72 mmol) and H-D,L-Phe(β-OH)-OMe,HCl gave compound **6a** as a light solid (0.27 g, 73%); mp: 130.0–133.0 °C; ^1^H NMR (400 MHz, CDCl_3_, δ): 2.56–2.61 (m, 1H, βCH), 2.72–2.79 (m, 2H, 2 × βCH), 2.86 (dd, *J* = 6.4 and 14.0 Hz, 1H, βCH), 3.55 (s, 4H, 2 × CH_2_), 3.61 (s, 6H, 2 × OCH_3_), 4.60–4.76 (m, 4H, 4 × αCH), 5.14 (d, *J* = 3.2 Hz, 1H, βCH), 5.21 (d, *J* = 3.2 Hz, 1H, βCH), 5.84–5.89 (m, 1H, NH), 5.94–5.99 (m, 1H, NH), 6.51–6.56 (m, 2H, Ar H), 6.82–6.89 (m, 4H, Ar H), 6.95–7.27 (m, 19H, 17 × Ar H, 2 × NH), 7.40–7.48 (m, 6H, Ar H), 7.66–7.69 (m, 3H, Ar H), 7.73–7.75 (m, 2H, Ar H); ^13^C NMR (100.6 MHz, CDCl_3_, δ): 37.25 (βCH_2_), 37.47 (βCH_2_), 43.58 (CH_2_), 43.63 (CH_2_), 52.61 (OCH_3_), 52. 67 (OCH_3_), 53.68 (αCH Phe), 53.97 (αCH Phe), 58.32 (αCH), 58.38 (αCH), 73.38 (βCH), 73.49 (βCH), 125.78 (CH), 125.84 (CH), 126.01 (CH), 126.09 (CH), 126.30 (CH), 126.38 (CH), 126.70 (CH), 126.76 (CH), 127.08 (2 × CH), 127.65 (CH), 127.68 (CH), 127.70 (CH), 127.95 (CH), 128.03 (CH), 128.08 (CH), 128.22 (CH), 128.25 (CH), 128.27 (CH), 128.30 (CH), 128.42 (CH), 128.45 (CH), 128.75 (CH), 128.81 (CH), 129.08 (CH), 129.15 (CH), 131.48 (C), 131.59 (C), 133.49 (2 × C), 133.52 (2 × C), 135.64 (C_i_ Phe), 135.90 (C_i_ Phe), 139.54 [C_i_ Phe(β-OH)], 139.68 [C_i_ Phe(β-OH)], 170.44 (C=O), 170.63 (C=O), 170.84 (C=O), 171.03 (C=O), 171.14 (C=O), 171.16 (C=O); HRMS (ESI) *m*/*z*: [M+H]^+^ calcd for C_31_H_31_N_2_O_5_^+^ 511.2228; found, 511.2237; [M+Na]^+^ calcd for C_31_H_30_N_2_NaO_5_^+^ 533.2047; found, 533.2061.

2-Naph-L-Ala-D,L-Phe(β-OH)-OMe (**6b**): 2-Naph-L-Ala-OH (**5b**) (0.44 g, 1.71 mmol) and H-D,L-Phe(β-OH)-OMe,HCl gave compound **6b** as a light oil, that spontaneously crystallized (0.33 g, 45%); mp: 112.0–115.0 ◦C; ^1^H NMR (400 MHz, CDCl_3_, δ): 1.01 (d, *J* = 7.2 Hz, 3H, βCH_3_), 1.24 (d, *J* = 6.8 Hz, 3H, βCH^3^), 3.68 (s, 2H, CH_2_), 3.69 (s, 2H, CH_2_), 3.70 (s, 3H, OCH_3_), 3.71 (s, 3H, OCH_3_), 4.45–4.56 (m, 2H, αCH), 4.80 (dd, *J* = 3.6 and 8.8. Hz, 1H, αCH), 4.85 (dd, *J* = 3.2 and 8.8 Hz, 1H, αCH), 5.25 (d, *J* = 3.6 Hz, 1H, βCH), 5.32 (d, *J* = 3.2 Hz, 1H, βCH), 6.20 (d, *J* = 7.6 Hz, 1H, NH), 6.35 (d, *J* = 7.6 Hz, 1H, NH), 7.20–7.35 (m, 14H, 12 × Ar H and 2 × NH), 7.42–7.50 (m, 4H, Ar H), 7.66 (d, *J* = 8.0 Hz, 2H, Ar H), 7.76–7.82 (m, 6H, Ar H); ^13^C NMR (100.6 MHz, CDCl_3_, δ): 18.12 (βCH_3_), 18.53 (βCH_3_), 43.52 (CH_2_), 43.56 (CH_2_), 48.78 (αCH), 48.88 (αCH), 52.56 (OCH_3_), 52.61 (OCH_3_), 58.10 (αCH), 58.37 (αCH), 73.43 (βCH), 73.48 (βCH), 125.66 (2 × CH), 125.87 (2 × CH), 125.97 (CH), 126.02 (CH), 126.30 (CH), 126.36 (CH), 127.05 (2 × CH), 127.66 (2 × CH), 127.67 (2 × CH), 127.85 (CH), 127.93 (CH), 128.20 (2 × CH), 128.27 (2 × CH), 128.30 (2 × CH), 128.74 (CH), 128.76 (CH), 131.62 (C), 131.64 (C),132.51 (C), 132.52 (C), 133.49 (2 × C), 139.65 (2 × C), 170.59 (C=O), 170.62 (C=O), 171.13 (C=O Naph), 171.14 (C=O Naph), 172.08 (C=O Ala), 172.12 (C=O Ala); HRMS (ESI) *m*/*z*: [M+H]^+^ calcd for C_25_H_27_N_2_O_5_^+^ 435.1915; found, 435.1912.

2-Naph-L-Phe-D,L-Ser-OMe (**6c**): 2-Naph-L-Phe-OH (**5a**) (0.36 g, 1.08 mmol) and H-D,L-Ser-OMe,HCl gave compound **6c** as a white solid (0.24 g, 51%), after a column chromatography (petroleum ether, ethyl acetate, mixtures of crescent polarity); mp: 166.0–168.0 °C; ^1^H NMR (300 MHz, CDCl_3_, δ): 2.92 (dd, *J* = 8.0 and 14.0 Hz, 1H, βCH Phe), 3.06 (dd, *J* = 5.9 and 14.0 Hz, 1H, βCH Phe), 3.58 (t, *J* = 6.5 Hz, 1H, OH), 3.65 (s, 2H, CH_2_ Naph), 3.73 (s, 3H, OCH_3_), 3.82–3.88 (m, 2H, βCH_2_ Ser), 4.54–4.59 (m, 1H, αCH Ser), 4.71–4.78 (m, 1H, αCH Phe), 6.37 (d, *J* = 7.8 Hz, 1H, NH Phe), 6.92–6.95 (m, 2H, 2 × H_o_ Phe), 7.00–7.05 (m, 2H, 2 × Hm Phe), 7.08–7.13 (m, 1H, H_p_ Phe), 7.21 (dd, *J* = 1.7 and 8.3 Hz, 1H, Ar H Naph), 7.25 (s, 1H, NH Ser), 7.46–7.52 (m, 12H, 2 × Ar H Naph), 7.58 (s, 1H, Ar H Naph), 7.73–7.76 (m, 2H, 2 × Ar H Naph), 7.80–7.83 (m, 1H, Ar H Naph); ^13^C NMR (75.5 MHz, CDCl_3_, δ): 37.84 (βCH_2_ Phe), 43.47 (CH_2_ Naph), 52.61 (OCH_3_), 54.48 (αCH Phe), 54.85 (αCH Ser), 62.51 (βCH_2_ Ser), 126.02 (CH Naph), 126.32 (CH Naph), 126.87 (CH_p_ Phe), 127.08 (CH Naph), 127.64 (CH Naph), 127.69 (CH Naph), 128.22 (CH Naph), 128.42 (CH_m_ Phe), 128.72 (CH Naph), 129.09 (CH_o_ Phe), 131.60 (C Naph), 132.50 (C Naph), 133.46 (C Naph), 135.79 (C_i_ Phe), 170.53 (C=O Ser), 171.17 (C=O Phe), 171.70 (C=O Naph); Anal. calcd for C_25_H_26_N_2_O_5_: C 69.11, H 6.03, N 6.45; found: C 68.52, H 6.485, N 7.151; HRMS (ESI) *m*/*z*: [M+H]^+^ calcd for C_25_H_27_N_2_O_5_^+^ 435.1915; found, 435.1914.

Synthesis of dehydrodipeptide derivatives (**7a**–**c**): DMAP (0.1 equiv.) was added to solutions of compounds **6a**–**c** in dry acetonitrile (1 M) followed by Boc_2_O (1.0 equiv.) under rapid stirring at room temperature. The reaction was monitored by ^1^H NMR until all the reactant had been consumed. Then TMG (2% in volume) was added, stirring was con-tinued and the reaction followed by ^1^H NMR. When all the reactant had been consumed, evaporation at reduced pressure gave a residue that was partitioned between ethyl acetate (50 mL) and KHSO_4_ (30 mL, 1 M). The organic phase was thoroughly washed with KHSO_4_ (1 M), NaHCO_3_ (1 M) and brine (3 × 30 mL, each), and dried with MgSO_4_. Removal of the solvent afforded compounds **7a**–**c**.

2-Naph-L-Phe-Z-ΔPhe-OMe (**7a**): 2-Naph-L-Phe-D,L-Phe(β-OH)-OMe (**6a**) (0.27 g, 0.53 mmol) gave compound **7a** as a light solid (0.16 g, 62%); mp: 197.0–200.0 °C; ^1^H NMR (400 MHz, DMSO-*d_6_*, δ): 2.84 (dd, *J* = 10.4 and 13.6 Hz, 1H, βCH), 3.13 (dd, *J* = 4.0 and 13.6 Hz, 1H, βCH), 3.59 (q, *J* = 14.0 Hz, 2H, CH_2_), 3.69 (s, 3H, OCH_3_), 4.72 (ddd, *J* = 4.0, 8.4 and 10.4 Hz, 1H, αCH), 7.16–7.25 (m, 6H, 5 × Ar H and βCH), 7.29–7.31 (m, 4H, Ar H), 7.42–7.49 (m, 2H, Ar H), 7.60–7.62 (m, 3H, Ar H), 7.73–7.77 (m, 2H, Ar H), 7.83–7.85 (m, 1H, Ar H), 8.53 (d, *J* = 8.4 Hz, 1H, NH), 9.92 (s, 1H, NH); ^13^C NMR (100.6 MHz, DMSO-*d_6_*, δ): 37.11 (βCH_2_), 42.15 (CH_2_ Naph), 52.17 (OCH_3_), 54.09 (αCH Phe), 125.41 (CH), 125.86 (αC ΔPhe), 125.94 (CH), 126.30 (CH), 127.18 (CH), 127.31 (CH), 127.40 (CH), 127.56 (CH), 128.03 (CH), 128.93 (CH), 129.23 (CH), 128.40 (CH), 130.02 (CH), 131.69 (C), 132.01 (CH), 132.01 (βCH ΔPhe), 132.89 (C), 133.93 (C-2 Naph), 137.76 (C_i_ Phe), 138.18 (C_i_ ΔPhe), 165.33 (C=O ΔPhe), 170.05 (C=O Naph), 171.47 (C=O Phe); HRMS (ESI) *m*/*z*: [M+H]^+^ calcd for C_31_H_29_N_2_O_4_^+^ 493.2122; found, 493.2128; [M+Na]^+^ calcd for C_31_H_28_N_2_NaO_4_^+^ 515.1941; found, 515.1949.

2-Naph-L-Ala-Z-ΔPhe-OMe (**7b**): 2-Naph-L-Ala-D,L-Phe(β-OH)-OMe (**6b**) (0.26 g, 0.60 mmol) gave compound **7b** as a light yellow solid (0.21 g, 84%); mp: 159.5–167.0 ◦C; ^1^H NMR (400 MHz, CDCl_3_, δ): 1.36 (d, *J* = 6.8 Hz, 3H, βCH_3_), 3.69 (s, 2H, CH_2_), 3.78 (s, 3H, OCH_3_), 4.70–4.77 (m, 1H, αCH), 6.24 (d, *J* = 7.2 Hz, 1H, NH), 7.28–7.33 (m, 4H, Ar H), 7.37 (s, 1H, βCH), 7.43–7.50 (m, 4H, Ar H), 7.67 (s, 1H, Ar H), 7.75–7.82 (m, 3H, Ar H), 8.04 (s, 1H, NH); ^13^C NMR (100.6 MHz, CDCl_3_, δ): 17.53 (βCH_3_), 43.51 (CH_2_), 49.10 (αCH), 52.59 (OCH_3_), 123.88 (αC), 126.01 (CH), 126.35 (CH), 127.03 (CH), 127.65 (CH), 127.67 (CH), 128.20 (CH), 128.53 (CH), 128.78 (CH), 129.54 (CH), 129.67 (CH), 131.64 (C), 132.52 (C), 133.03 (βCH), 133.41 (C), 133.49 (C), 165.25 (C=O ΔPhe), 170.83 (C=O Ala), 171.31 (C=O Naph); HRMS (ESI) *m*/*z*: [M+H]_+_ calcd for C_25_H_25_N_2_O_4_^+^ 417.1809; found, 417.1806; [M+Na]^+^ calcd for C_25_H_24_N_2_NaO_4_^+^ 439.1628; found, 439.1626.

2-Naph-L-Phe-ΔAla-OMe (**7c**): 2-Naph-L-Phe-D,L-Ser-OMe (**6c**) (0.30 g, 0.69 mmol) gave compound **7c** as a white solid (0.21 g, 72%); mp: 65.0–67.0 °C; ^1^H NMR (400 MHz, CDCl_3_, δ): 2.99–3.01 (m, 2H, βCH_2_), 3.73 (s, 2H, CH_2_), 3.81 (s, 3H, OCH_3_), 4.71–4.76 (m, 1H, αCH), 5.89 (s, 1H, βCH), 5.99 (d, *J* = 7.6 Hz, 1H, NH), 6.55 (s, 1H, βCH), 6.89 (d, *J* = 7.2 Hz, 2H, H_o_ Phe), 7.02 (t, *J* = 7.4 Hz, 2H, H_m_ Phe), 7.11 (t, *J* = 7.4 Hz, 1H, H_p_ Phe), 7.23–7.29 (m, 1H, H-3 Naph), 7.50–7.54 (m, 2H, Ar H Naph), 7.63 (s, 1H, H-1 Naph), 7.78–7.81 (m, 2H, Ar H Naph), 7.84.7.87 (m, 1H, Ar H Naph), 8.24 (s, 1H, NH); ^13^C NMR (100.6 MHz, CDCl_3_, δ): 37.29 (βCH_2_), 43.62 (CH_2_ Naph), 52.91 (OCH_3_), 54.94 (αCH Phe), 109.37 (βCH_2_), 126.11 (CH Naph), 126.38 (CH Naph), 126.95 (C_p_ Phe), 127.09 (CH-3 Naph), 127.67 (CH Naph), 127.71 (CH Naph), 128.34 (CH-1 Naph), 128.56 (C_m_ Phe), 128.91 (CH Naph and C_o_ Phe), 130.58 (αC), 131.45 (C-2 Naph), 132.57 (C-4a Naph), 133.53 (C-8a Naph), 135.55 (C_i_ Phe), 163.95 (C=O ΔAla), 169.41 (C=O Phe), 171.19 (C=O Naph); HRMS (ESI) *m*/*z*: [M+H]^+^ calcd for C_25_H_25_N_2_O_4_^+^ 417.18088; found, 417.18060; [M+Na]^+^ calcd for C_25_H_24_N_2_NaO_4_^+^ 439.16283; found, 439.16256.

Synthesis of 1-naphthoyl dehydrodipeptides (**10a**,**b**): The methyl ester of the peptide (1.1 equiv.) was dissolved in DCM (5 mL) and put in an ice bath. Triethylamine (2.2 equiv.) was added and, slowly, 1-naphthoyl chloride. The mixture was left stirring at rt overnight (~18 h). The mixture was filtered. Evaporation at reduced pressure gave a residue that was partitioned between ethyl acetate (50 mL) and KHSO_4_ (30 mL, 1 M). The organic phase was thoroughly washed with KHSO_4_ (1 M), NaHCO_3_ (1 M) and brine (3 × 30 mL, each), and dried with MgSO_4_. Removal of the solvent afforded compounds **10a**,**b**.

1-Nap-L-Phe-Z-ΔPhe-OMe (**10a**): H-L-Phe-Z-ΔPhe-OMe.TFA (**9a**) (0.39 g, 0.90 mmol) and 1-naphthoyl chloride gave compound **10a** as a white solid (0.38 g, 88%); mp: 197.0–198.0 °C; ^1^H NMR (400 MHz, DMSO-*d_6_*, δ): 2.95–3.02 (dd, *J* = 11.2 and 2.4 Hz, 1H, βCH_2_), 3.23–3.28 (dd, *J* = 3.6 and 10.4 Hz, 1H, βCH_2_ Phe), 3.74 (s, 3H, OCH_3_), 4.99–5.06 (m, 1H, αCH Phe), 7.25–7.29 (m, 1H, Ar H), 7.32–7.41 (m, 7H, Ar H and βCH ΔPhe), 7.42–7.46 (m, 3H, Ar H), 7.48–7.53 (m, 2H, Ar H), 7.74–7.77 (m, 2H, Ar H), 7.87 (d, *J* = 8.4 Hz, 1H, Ar H), 7.93 (d, *J* = 8.0 Hz, 1H, Ar H), 7.98 (d, *J* = 8.4 Hz, 1H, Ar H), 8.86 (d, *J* = 8.4 Hz, 1H, NH Phe), 10.02 (s, 1H, NH ΔPhe); ^13^C NMR (100.6 MHz, DMSO-*d_6_*, δ): 36.63 (βCH_2_ Phe), 52.25 (OCH_3_), 54.85 (αCH Phe), 124.82 (CH), 125.21 (CH), 125.54 (CH), 125.96 (αC), 126.12 (CH), 126.40 (CH), 126.42 (CH), 127.99 (CH), 128.15 (CH), 128.60 (CH), 129.31 (CH), 129.53 (CH), 129.68 (C), 129.77 (CH), 130.14 (CH), 132.10 (βCH ΔPhe), 132.99 (C), 133.31 (C), 134.38 (C),138.20 (C), 165.41 (C=O), 168.70 (C=O), 171.57 (C=O); Anal. calcd for C_30_H_26_N_2_O_4_: C 75.30, H 5.48, N 5.85; found: C 74.84, H 5.57, N 5.92.

1-Nap-L-Phe-Z-ΔAbu-OMe (**10b**): H-L-Phe-Z-ΔAbu-OMe.TFA (**9b**) (0.53 g, 1.4 mmol) and 1-naphthoyl chloride gave compound **10b** as a white solid (0.47 g, 80%); mp: 164.0–165.0 °C; ^1^H NMR (400 MHz, CDCl_3_, δ): 1.70 (d, *J* = 7.2 Hz, 3H, γCH_3_ ΔAbu), 3.22–3.25 (m, 1H, βCH_2_), 3.36–3.40 (m, 1H, βCH_2_ Phe), 3.73 (s, 3H, OCH_3_), 5.22 (q, *J* = 7.6 Hz, 1H, αCH Phe), 6.74–6.83 (m, 2H, NH Phe and βCH ΔAbu), 7.28–7.41 (m, 6H, Ar H), 7.43–7.52 (m, 3H, Ar H), 7.84 (d, *J* = 8.0 Hz, 2H, Ar H), 7.89 (d, *J* = 8.0 Hz, 1H, Ar H), 8.07 (d, *J* = 8.4 Hz, 1H, NH ΔPhe); ^13^C NMR (100.6 MHz, CDCl_3_, δ): 14.42 (γCH_3_ ΔAbu), 37.78 (βCH_2_ Phe), 52.30 (OCH_3_), 54.87 (αCH Phe), 124.62 (CH), 125.18 (CH), 125.39 (CH), 125.92 (C), 126.41 (CH), 127.11 (CH), 127.19 (CH), 128.25 (CH), 128.77 (CH), 129.41 (CH), 129.99 (C), 131.04 (CH), 133.21 (C), 133.58 (C), 134.69 (βCH ΔAbu), 136.38 (C), 164.53 (C=O), 169.38 (C=O), 169.74 (C=O); HRMS (micrO-TOF) *m*/*z*: [M+H]+ calcd. for C_25_H_25_N_2_O_4_^+^ 417.1814; found, 417.1809.

Synthesis of the N-capped C-deprotected dehydrodipeptides (**1a**–**c**, **2a**,**b**): The N-protected dehydrodipeptide (**7a**–**c** and **10a**,**b**) was dissolved in 1,4-dioxane (10 mL) and NaOH (1 M) (1.5 equiv.). The reaction was followed by TLC until no starting material was detected. The organic solvent was removed under reduced pressure, and the reaction mixture was acidified to pH 3 with KHSO_4_ (1 M). The solid formed was filtered, affording compounds **1a**–**c**, **2a**,**b**.

2-Naph-L-Phe-Z-ΔPhe-OH (**1a**): 2-Naph-L-Phe-Z-ΔPhe-OMe (**7a**) (0.34 g, 0.69 mmol) gave compound **1a** as a white solid (0.31 g, 94%); mp: 165.0–167.0 °C; ^1^H NMR (400 MHz, DMSO-*d_6_*, δ): 2.78–2.84 (m, 1H, βCH), 3.11–3.16 (m, 1H, βCH), 3.51–3.59 (m, 2H, CH_2_), 4.68–4.74 (m, 1H, αCH), 7.15–7.24 (m, 5H, Ar H), 7.27–7.30 (m, 5H, Ar H and βCH), 7.42–7.48 (m, 2H, Ar H), 7.58–7.60 (m, 3H, Ar H), 7.72–7.76 (m, 2H, Ar H), 7.82–7.85 (m, 1H, Ar H), 8.47 (d, *J* = 8.4 Hz, 1H, NH), 9.73 (s, 1H, NH); ^13^C NMR (100.6 MHz, DMSO-*d_6_*, δ): 37.16 (βCH_2_), 42.18 (CH_2_), 54.14 (αCH), 125.43 (CH), 125.95 (CH), 126.27 (CH), 126.54 (αC), 127.19 (CH), 127.33 (CH), 127.41 (CH), 127.57 (CH), 128.02 (CH), 128.46 (CH), 129.18 (CH), 129.25 (CH), 129.93 (CH), 131.70 (C), 131.93 (βCH), 132.89 (C), 133.54 (C), 133.94 (C-2), 137.85 (C_i_ Phe), 166.19 (C=O), 170.06 (C=O), 171.16 (C=O); HRMS (ESI) *m*/*z*: [M+Na]^+^ calcd for C_30_H_26_N_2_NaO_4_^+^ 501.17848; found, 501.17831; [M+H]^+^ calcd for C_30_H_27_N_2_O_4_^+^ 479.1965; found, 479.1963.

2-Naph-L-Ala-Z-ΔPhe-OH (**1b**): 2-Naph-L-Ala-Z-ΔPhe-OMe (**7b**) (0.18 g, 0.42 mmol) gave compound **1b** as a light solid (0.13 g, 76%); mp: 161.0–163.0 °C; ^1^H NMR (400 MHz, DMSO-*d_6_*, δ): 1.30 (d, *J* = 7.2 Hz, 3H, βCH_3_), 3.66 (s, 2H, CH_2_), 4.46 (quint, *J* = 7.2 Hz, 1H, αCH), 7.24–7.31 (m, 4H, 3 × Ar H and βCH), 7.43–7.49 (m, 3H, Ar H), 7.59–7.62 (m, 2H, Ar H), 7.76 (s, 1H, Ar H), 7.81–7.87 (m, 3H, Ar H), 8.40 (d, *J* = 7.2 Hz, 1H, NH Ala), 9.50 (s, 1H, NH ΔPhe), 12.68 (brs, 1H, CO_2_H); ^13^C NMR (100.6 MHz, DMSO-*d_6_*, δ): 17.84 (βCH_3_), 42.01 (CH_2_), 48.32 (αCH), 125.44 (CH), 125.98 (CH), 126.60 (C), 127.30 (CH), 127.35 (CH), 127.43 (CH), 127.49 (CH), 127.71 (CH), 128.37 (CH), 129.07 (CH), 129.93 (CH), 131.73 (βCH), 131.74 (C), 132.95 (C), 133.62 (C), 134.05 (C), 166.23 (C=O ΔPhe), 169.88 (C=O Naph), 171.93 (C=O Ala); HRMS (ESI) *m*/*z*: [M+H]^+^ calcd for C_24_H_23_N_2_O_4_^+^ 403.1652; found, 403.1659.

2-Naph-L-Phe-ΔAla-OH (**1c**): 2-Naph-L-Phe-ΔAla-OMe (**7c**) (0.21 g, 0.50 mmol) gave compound **1c** as a white solid (0.13 g, 65%); mp: 159.0–160.0 °C; ^1^H NMR (400 MHz, DMSO-*d_6_*, δ): 2.82 (dd, *J* = 10.4 and 13.6 Hz, 1H, βCH), 3.08 (dd, *J* = 4.0 and 13.6 Hz, 1H, βCH), 3.52–3.61 (m, 2H, CH_2_), 4.72–4.76 (m, 1H, αCH), 5.73 (s, 1H, βCH), 6.31 (s, 1H, βCH), 7.16–7.23 (m, 4H, 2 × H_m_ Phe, H_p_ Phe, Ar H Naph), 7.27–7.29 (m, 2H, 2 × H_o_ Phe), 7.44–7.48 (m, 2H, 2 × Ar H Naph), 7.59 (s, 1H, H-1 Naph), 7.73 (d, *J* = 8.4 Hz, 1H, Ar H Naph), 7.77 (dd, *J* = 2.0 and 7.8 Hz, 1H, Ar H Naph), 8.56 (dd, *J* = 1.6 and 7.6 Hz, 1H, Ar H Naph), 8.56 (d, *J* = 8.0 Hz, 1H, NH Phe), 9.28 (s, 1H, NH ΔAla), 12.80 (brs, 1H, CO_2_H); ^13^C NMR (100.6 MHz, DMSO-*d_6_*, δ): 36.85 (βCH_2_), 42.07 (CH_2_ Naph), 54.67 (αCH Phe), 108.10 (βCH_2_), 125.46 (CH Naph), 125.98 (CH Naph), 126.27 (C_p_ Phe), 127.17 (CH-1 Naph), 127.32 (CH), 127.41 (CH), 127.48 (2 × CH), 127.99 (CH), 129.24 (C_o_ Phe), 131.70 (C Naph), 132.80 (αC), 132.89 (C Naph), 133.69 (C Naph), 137.64 (C_i_ Phe), 164.79 (C=O ΔAla), 170.27 (C=O Naph), 170.92 (C=O Phe); HRMS (ESI) *m*/*z*: [M+H]^+^ calcd for C_24_H_23_N_2_O_4_^+^ 403.16578; found, 403.16522; [M+Na]^+^ calcd for C_24_H_22_N_2_NaO_4_^+^ 425.1477; found, 425.14716; [M+K]^+^ calcd for C_24_H_22_N_2_KO_4_^+^ 441.1217; found, 441.1210.

1-Nap-L-Phe-Z-ΔPhe-OH (**2a**): 1-Nap-L-Phe-Z-ΔPhe-OMe (**10a**) (0.32 g, 0.67 mmol) gave compound **2a** as a white solid (0.29 g, 94%); mp: 181.0–182.0 °C; ^1^H NMR (400 MHz, DMSO-*d_6_*, δ): 2.92–2.98 (dd, *J* = 11.6 and 2.4 Hz, 1H, βCH_2_ Phe), 3.24–3.29 (dd, *J* = 3.2 and 10.4 Hz, 1H, βCH_2_ Phe), 4.97–5.03 (m, 1H, αCH Phe), 7.25–7.36 (m, 7H, Ar H and βCH ΔPhe), 7.38–7.51 (m, 6H, Ar H), 7.61 (d, *J* = 6.8 Hz, 2H, Ar H), 7.80 (d, *J* = 8.4 Hz, 1H, Ar H), 7.91 (d, *J* = 8.0 Hz, 1H, Ar H), 7.96 (d, *J* = 7.6 Hz, 1H, Ar H), 8.87 (d, *J* = 8.4 Hz, 1H, NH Phe), 9.74 (s, 1H, NH ΔPhe); ^13^C NMR (100.6 MHz, DMSO-*d_6_*, δ): 36.65 (βCH_2_ Phe), 55.09 (αCH Phe), 124.85 (CH), 125.17 (CH), 125.60 (CH), 126.12 (CH), 126.34 (CH), 126.42 (CH), 127.95 (CH), 128.14 (CH), 128.24 (CH), 128.44 (CH), 128.64 (C), 129.34 (CH), 129.65 (C), 129.71 (CH), 129.76 (CH), 132.98 (C), 134.53 (C), 134.67 (C), 138.37 (C), 166.14 (C=O), 170.93 (C=O), 173.33 (C=O); HRMS (micrOTOF) *m*/*z*: [M+Na]^+^ calcd. for C_29_H_24_N_2_NaO_4_+ 487.1634; found, 487.1629.

1-Nap-L-Phe-Z-ΔAbu-OH (**2b**): 1-Nap-L-Phe-Z-ΔAbu-OMe (**10b**) (0.33 g, 0.79 mmol) gave compound **2b** as a white solid (0.31 g, 97%); mp: 200.0–201.0 °C; ^1^H NMR (400 MHz, DMSO-*d_6_*, δ): 1.69 (d, *J* = 7.2 Hz, 3H, γCH_3_ ΔAbu), 2.92–2.99 (dd, *J* = 11.2 and 2.4 Hz, 1H, βCH_2_), 3.20–3.25 (dd, *J* = 4.0 and 10.0 Hz, 1H, βCH_2_ Phe), 4.97–5.03 (m, 1H, αCH Phe), 6.62 (q, *J* = 7.2 Hz, 1H, γCH ΔAbu), 7.11 (dd, *J* = 2.8 and 6.0 Hz, 1H, Ar H), 7.18–7.31 (m, 6H, Ar H), 7.56 (s, 1H, Ar H), 7.23–7.27 (m, 1H, Ar H), 7.30–7.33 (m, 2H, Ar H), 7.39–7.43 (m, 4H, Ar H), 7.46–7.52 (m, 2H, Ar H), 7.78 (d, *J* = 8.4 Hz, 1H, Ar H), 7.91 (d, *J* = 8.0 Hz, 1H, Ar H), 7.96 (d, *J* = 8.0 Hz, 1H, Ar H), 8.76 (d, *J* = 8.4 Hz, 1H, NH Phe), 9.39 (s, 1H, NH ΔAbu), 12.55 (brs, 1H, CO_2_H); ^13^C NMR (100.6 MHz, DMSO-*d_6_*, δ): 13.79 (γCH_3_ ΔAbu), 37.32 (βCH_2_ Phe), 54.64 (αCH Phe), 124.83 (CH), 125.07 (CH), 125.54 (CH), 126.11 (CH), 126.32 (CH), 126.42 (CH), 127.95 (CH), 128.10 (CH), 128.20 (αC ΔAbu), 129.33 (CH), 129.64 (C), 129.67 (CH), 132.18 (βCH ΔAbu), 132.97 (C), 134.57 (C), 138.21 (C), 165.55 (C=O), 168.50 (C=O), 170.10 (C=O); HRMS (micrOTOF) *m*/*z*: [M+Na]^+^ calcd for C_24_H_22_N_2_NaO_4_^+^ 425.1477; found, 425.1488.

Self-assembly: All solutions were made up with ultra-filtered (18 MΩ) water from a Barnstead Nanopure system (Thermofisher, Waltham, MA, USA). Phosphate buffer was prepared from sodium dihydrogen phosphate monohydrate (NaH_2_PO_4_·H_2_O, Fluka BioChemika, Honeywell, Charlotte, NC, USA) and sodium phosphate dibasic dodecahydrate (Na_2_HPO_4_·12H_2_O, Fluka, Honeywell, Charlotte, NC, USA) with a final concentration of 0.1 M and pH 6.00, 7.19 or 8.06 (Mettler Toledo FiveEasy pH Meter, Merck, Darmstadt, Germany). *D*-glucono-δ-lactone (GdL, Sigma, St. Louis, MO, USA), aqueous NaOH 1 M and HCl 0.1 M were used.

Self-assembly with buffer: Compounds were weighed into sample vials. Buffer was added and the mixture was sonicated at room temperature, followed by heating to 80 °C under magnetic stirring. Solutions were left to cool to room temperature for 24 h.

Self-assembly with GdL: Compounds were weighed into sample vials. Water was added and the suspension was adjusted to pH circa 10 with aqueous NaOH 1 M. The mixture was briefly sonicated at room temperature and added into a vial with the weighed amount of GDL. The mixture was stirred (1000 rpm during 10 s) and left standing at room temperature overnight. The pH of the hydrogel was measured with pH paper.

Circular dichroism: The CD spectra were recorded at 20 °C on a Chirascan spectropolarimeter (AppliedPhotophysics, Leatherhead, Surrey, UK). Peptide hydrogels were loaded into 0.1 mm quartz cells. Spectra display absorbance <2 at any measured point with 0.5 nm step, 1 nm bandwidth and 1 s collection time per step, taking three averages. The post-acquisition smoothing tool from Chirascan software (Version 4.13) was used to remove random noise elements from the averaged spectra. A residual plot was generated for each curve in order to check for spectral distortion effects during the smoothing process. CD data were normalized to molar mean residue ellipticity.

Scanning transmission electron microscopy: STEM experiments were performed using an ultra-high resolution field emission gun scanning electron microscopy (FEG-SEM), NOVA 200 Nano SEM, FEI Company, Hillsboro, Oregon, USA (SEMAT/UM), operated at 15 and 18.5 kV, using a STEM detector (PNDetector, Munich, Germany). Cu-C grids (S160-4 AGAR) were immersed in the peptide hydrogels. The grid was then allowed to dry at room temperature.

Transmission electron microscopy: TEM experiments were performed using a Philips CM20 (FELMI-ZFE, Graz, Austria) transmission electron microscope operating at 200 kV. Diluted solutions, 5 times more diluted than the measured cgc, were prepared in conditions similar to those used for preparation of the hydrogels. The shiny side of 300 mesh Cu grids coated with a carbon film (Agar Scientific, Stansted, Essex, UK) was placed over a drop of dehydrodipeptide solution (**1a** and **1c**) during 1 min; the excess solution at the sides of the grid was carefully cleaned. One drop of the peptide solution (**1b**, **2a** and **2b**) was placed on the shiny side of 300 mesh Cu grids coated with a carbon film (Agar Scientific, Stansted, Essex, UK) for 1 min; the excess solution at the sides of the grid was carefully cleaned. In all cases, the shiny side of the grid was placed over a drop of aqueous uranyl acetate (1 wt%) (Agar Scientific, Stansted, Essex, UK) for 1 min. The excess at the sides of the grid was cleaned very carefully and the grid was then allowed to dry at room temperature.

Rheology: Rheology experiments were carried out using a PaarPhysica MCR300 rheometer, Graz, Austria, equipped with a TEK 350-C plate with TC20/EDT/TEK temperature control and a cylindrical plunger with a diameter of 1 cm. Penetration tests on gelled samples were preferred to dynamic shear rheology performed on gelling samples in the rheometer. This is because very long (within 24 h) gel kinetics of samples would jeopardize the practicality of the study, which focuses on the viscoelastic comparison between gels and not on the gelation properties. The hydrogels were prepared in soda glass specimen tubes (Samco, Warwickshire, UK), which also served as the cups for the rheological measurements. The vials had diameters of 25 mm and the measurements were performed till a gap of 1.5 mm to the bottom of the vials was reached, avoiding significant end effects. The normal force was measured as a function of penetration distance in the gel. Young’s moduli were computed neglecting any plunger buoyancy effect, following the approach of Oakenfull et al. [40]. The slope and associated standard error of the linear regime of the stress–strain responses (Young’s moduli) and the power law exponents for the concentration regimes in **2b** were determined using OriginPro 8 software. Strain and stress at break correspond to the location of the first maximum in the stress–strain curves followed by a deep decrease in stress.

Spectroscopic measurements: Fluorescence measurements were performed using a Fluorolog 3 spectrofluorimeter (Kyoto, Japan), equipped with double monochromators in both excitation and emission, Glan-Thompson polarizers and a temperature-controlled cuvette holder. Fluorescence emission and excitation spectra were corrected for the instrumental response of the system.

Molecular dynamics (MD) simulations: The α,β-dehydroamino acids, ΔzPhe, ΔAla and ΔzAbu and Napthtalene were parameterized and validated in a previous work by some of the authors [29], from bonded and non-bonded parameters based on equivalent encoded amino acids of the GROMOS 54a7 force field [45]. The N-protected groups 2-naphthylacetyl (2-Naph) and 1-naphthoyl (1-Nyl) were parameterized based on the Naproxen group also used previously [29]. Topology files for 2-Naph, 1-Nyl, Npx, ΔzPhe, ΔAla and ΔzAbu are available upon request.

The initial molecular structure of the dehydrodipeptides was built with the program PyMOL [45]. MD simulations of dehydrodipeptides monomers were performed in octahedral boxes of SPC water with a hydration layer of at least 1.5 nm between the peptide and the periodic boundary conditions in all directions [46,47]. Boxes were made neutral with the addition of one Na^+^ ion. Each system was subjected to 12,000 steps of energy minimization with the steepest descent algorithm. Then, 100 ns of MD simulations were run, 40 ns of equilibration and 60 ns of production. MD simulations were run with the GROMACS 4.5.4 software package [48,49,50]. In all MD simulations, the system was maintained at constant temperature and pressure, 310 K and 1 atm, respectively, with the Berendsen thermostat and barostat methods, with ΔT = 0.10 ps and ΔP = 1.0 ps [51]. The SETTLE algorithm was used to constrain bond lengths and angles of water molecules, while the bond lengths and angles of peptides were constrained with the LINCS algorithm, which allowed the use of a 2 fs time step [52,53]. For the treatment of long-range interactions, we used the reaction field method, with a cutoff of 1.4 nm and a dielectric constant of 54 (corresponding to SPC water). The van der Waals interactions were truncated with a twin-range cutoff of 0.8 and 1.4 nm. The conformation population was computed with a clustering algorithm through a single-linkage method by first fitting the heavy atoms on the dehydrodipeptides and then analyzing the RMSD matrix vs. time to determine the most likely conformations for each peptide. The MD procedure used to simulate the dimers was similar to that used for the monomers. In this case, the dimers were solvated with SPC water in cubic boxes of 5 × 5 × 5 nm neutralized with two Na^+^ ions; the same energy minimization, equilibration and production run (40 + 60 ns) was also followed for the dimers. The aggregation analysis was done by monitoring the distance between the centers of mass of the peptides in the box vs. time for 60 ns and used to populate 0.1 nm bin histograms.

The most likely conformations for each molecule were also studied using quantum chemical DFT calculations. DFT calculations were performed with the program Gaussian 09 with the BLYP-D3 Hamiltonian that has been found to yield improved geometries in π–π stacked systems [54,55,56]. Calculations were performed with the SMD implicit water solvent with the def2-TVZP basis set.

## Figures and Tables

**Table 1:** Critical gelation concentration (CGC), gel-sol phase-transition pH (pHgs) and hydrophobicity for hydrogelators **1a**–**c** and **2a**,**b**.

Hydrogelator	CGC				miLogP ^2^
	wt%	mM	NaOH 1 M:GDL (%*v*/*v*; wt%) ^1^	pH	
2-Naph-L-Phe-ΔPhe-OH, **1a**	0.14 ^3^	2.93	Phosphate buffer	8	5.01
	0.5 ^4^	10.5	1.25:0.3	6	-
2-Naph-L-Ala-ΔPhe-OH, **1b**	0.5 ^4^	12.4	4.0:0.59	6	3.55
2-Naph-L-Phe-ΔAla-OH, **1c**	0.6 ^4^	14.9	1.9:1.5	4	3.30
1-Nap-L-Phe-ΔPhe-OH, **2a**	0.07 ^4^	1.51	4.0:0.75	5	4.89
-	0.4 ^4^	4.31	4.0:0.76	6	-
1-Nap-L-Phe-ΔAbu-OH, **2b**	0.2 ^4^	5.12	4.0:0.84	5	3.44
-	0.4 ^4^	10.3	4.0:0.65	6	-

^1^ % *v*/*v* of added NaOH 1 M and % (*m*/*v*) of added GDL. ^2^ miLogP is the logP value (octanol-water partition coefficient), a molecular descriptor of hydrophobicity/lipophilicity, calculated by the online resource molinspiration properties calculator (https://www.molinspiration.com/services, accessed on 4 May 2023). ^3^ cooling on-standing to rt a hot (80 °C) solution of hydrogelator in phosphate buffer (0.1 M, pH 8.0). ^4^ pH dropping with added GDL.

**Table 2:** Rheological properties of the hydrogels of peptides **1a**,**b** and **2a**,**b**.

Compound			Strain ^1^ [%]	Stress ^1^ [Pa]	Young Modulus ^2^ [Pa]	Shear Modulus ^3^ [kPa]
	wt%	mM				
**1a**	0.30	6.27	34	1249	14,567 ± 53.8	4.90 ± 0.02
**1b**	0.69	17.15	29	1800	13,013.8 ± 105.3	4.34 ± 0.03
**2a**	0.10	2.15	22	1969	25,625.3 ± 98.5	8.54 ± 0.03
**2b**	0.81	20.13	27	15,071	14,358.7 ± 106.1	4.79 ± 0.03

^1^ Values taken at the breaking point. ^2^ Determined from the slope of the linear regime of the stress–strain response curve showing up right after the non-linear strain regime for probe accommodation to the gel’s surface. ^3^ Determined from Young’s modulus (G = E/3).

## Supplementary Materials

The following supporting information can be downloaded at: https://www.mdpi.com/article/10.3390/gels9060464/s1, Figure S1: Phase diagrams for hydrogelators obtained by addition of GDL to peptide solutions adjusted to pH circa 10 with NaOH 1M: (A) **1b**; (B) **1c**. *approximate pH of the gel (measured with indicator paper). Figure S2. Phase diagrams for dehydrodipeptide **2a**, obtained by addition of GdL to alkaline solutions of this peptide (4 %(*v*/*v*) of NaOH 1 m); (A) Correlation between pH and peptide concentration; (B) Correlation between equivalents of GdL and peptide concentration. Figure S3. Phase diagrams for dehydrodipeptide **2b**, obtained by addition of GdL to alkaline solutions of this peptide (4 %(*v*/*v*) of NaOH 1 m); (A) Correlation between pH and peptide concentration (arrows represent the drop in pH by addition of more GdL to the same solution); (B) Correlation between equivalents of GdL and peptide concentration. Figure S4. UV-VIS absorption spectra for hydrogelators **1a**–**c** and **2a**,**b** in gel phase at the same concentration and pH as that deployed for the CD measurements: (A) gels **1a**–**c**; (B) gels **2a**,**b**. UV-VIS absorption spectra for hydrogelators **1a**–**c** and **2a**,**b** in solution, at concentrations well below the CGC: (C) gels **1a**–**c**; (D) gels **2a**,**b**. Figure S5. STEM images: (A,B) **1a** (gel at 0.5 wt% in phosphate buffer, pH 8), scale (A) 2 μm, (B) 500 nm; (C) **1b** [gel at 0.77 wt%] scale 1 μm; (D) **1c** [gel at 0.6 wt%], scale 1 μm. Figure S6. TEM images: (A,B,C) **1b** (0.65 wt%), scale (A) 2 μm; (B,C) 500 nm; (D,E) **2a** (0.014 wt%), scale 100 nm; (F) **2b** (0.040 wt%), scale 500 nm. Figure S7. Stress-strain response curves for penetration tests for hydrogels at 25 μm s^−1^ plunger speed.: (A) gel **2a**; (B) gel **2b**; (C) Zoom of the linear regime of the stress-strain response curve of compounds **2b**.
