# Supplementary material for: Unveiling the Role of Capping Groups in Naphthalene N-Capped Dehydrodipeptide Hydrogels
Source: Gels. 2023 Jun 6;9(6):464. doi: 10.3390/gels9060464 (PMC10298498; doi:10.3390/gels9060464)
Supplement: Supplementary file 1 [file gels-09-00464-s001.zip › gels-2414022-supplementary.pdf]

gels

MDPI

Article

Unveiling the Role of Capping Groups in Naphthalene N-Capped Dehydrodipeptide Hydrogels

Helena Vilaça [1,2], André Carvalho [1], Tarsila Castro [3], Elisabete M. S. Castanheira [4], Loic Hilliou [5], Ian Hamley [6], Manuel Melle-Franco [7], Paula M. T. Ferreira [1,*] and José A. Martins [1,*]

[1] Centre of Chemistry, University of Minho, Campus de Gualtar, 4710-057 Braga, Portugal; hvilaca@citeve.pt (H.V.); andrefcarvalho95@gmail.com (A.C.)
[2] Department of Chemistry and Biotechnology, Technological Centre for the Textile and Clothing Industries of Portugal, 4760-034 Vila Nova de Famalicão, Portugal
[3] Centre of Biological Engineering, University of Minho, Campus de Gualtar, 4710-057 Braga, Portugal; castro.tarsila@ceb.uminho.pt
[4] Physics Centre of Minho and Porto Universities (CF-UM-UP), University of Minho, Campus de Gualtar, 4710-057 Braga, Portugal; ecoutinho@fisica.uminho.pt
[5] Institute for Polymers and Composites, Department of Polymer Engineering, University of Minho, Campus de Azurém, 4800-058 Guimarães, Portugal; loic@dep.uminho.pt
[6] Department of Chemistry, University of Reading, Whiteknights, P.O. Box 224, Reading RG6 6AD, UK; i.w.hamley@reading.ac.uk
[7] CICECO-Aveiro Institute of Materials, Department of Chemistry, University of Aveiro, 3810-193 Aveiro, Portugal; manuelmelle.research@gmail.com
* Correspondence: pmf@quimica.uminho.pt (P.M.T.F.); jmartins@quimica.uminho.pt (J.A.M.)

Supplementary Materials

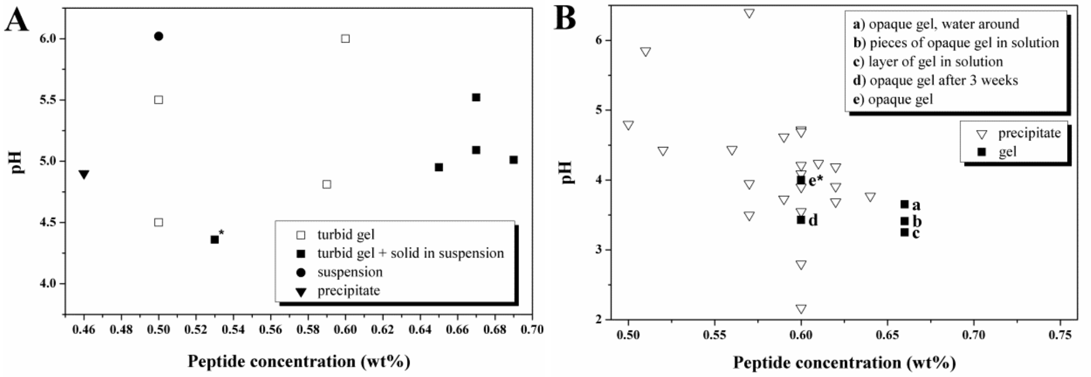

Figure S1. Phase diagrams for hydrogelators obtained by addition of GDL to peptide solutions adjusted to pH circa 10 with NaOH 1M: A) **1b**; B) **1c**. *approximate pH of the gel (measured with indicator paper).

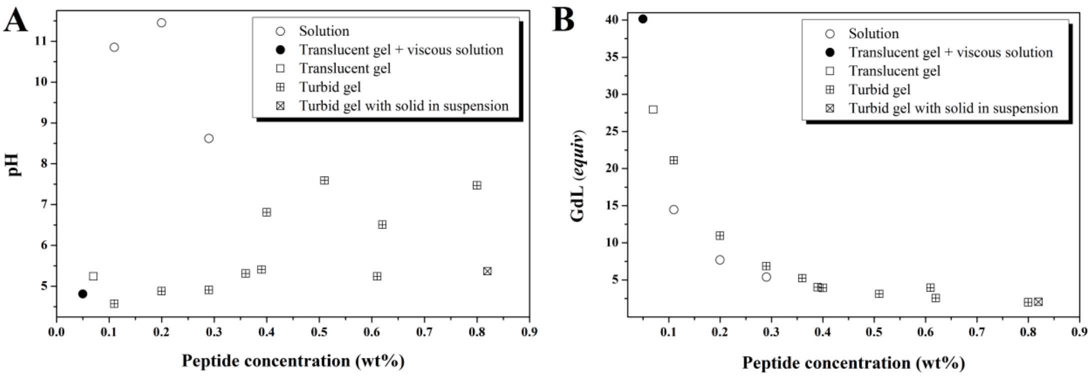

Figure S2. Phase diagrams for dehydrodipeptide **2a**, obtained by addition of GdL to alkaline solutions of this peptide (4 %(v/v) of NaOH 1 M); A) Correlation between pH and peptide concentration; B) Correlation between equivalents of GdL and peptide concentration.

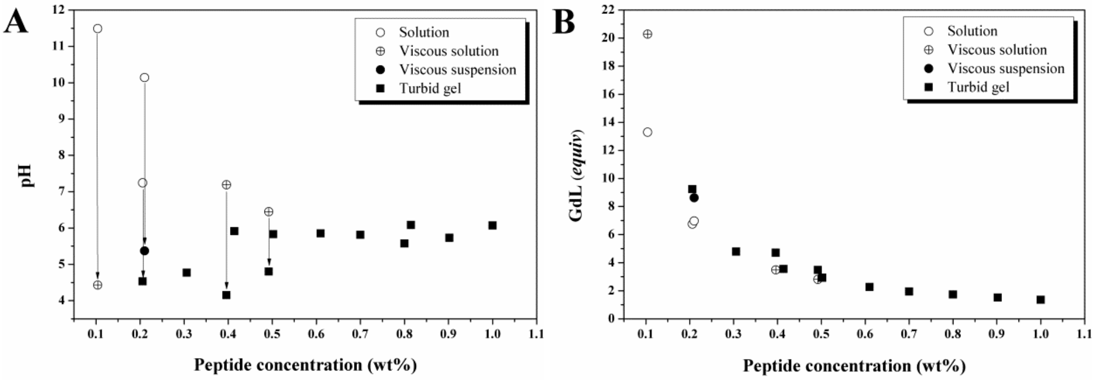

Figure S3. Phase diagrams for dehydrodipeptide **2b**, obtained by addition of GdL to alkaline solutions of this peptide (4 %(v/v) of NaOH 1 M); A) Correlation between pH and peptide concentration (arrows represent the drop in pH by addition of more GdL to the same solution); B) Correlation between equivalents of GdL and peptide concentration.

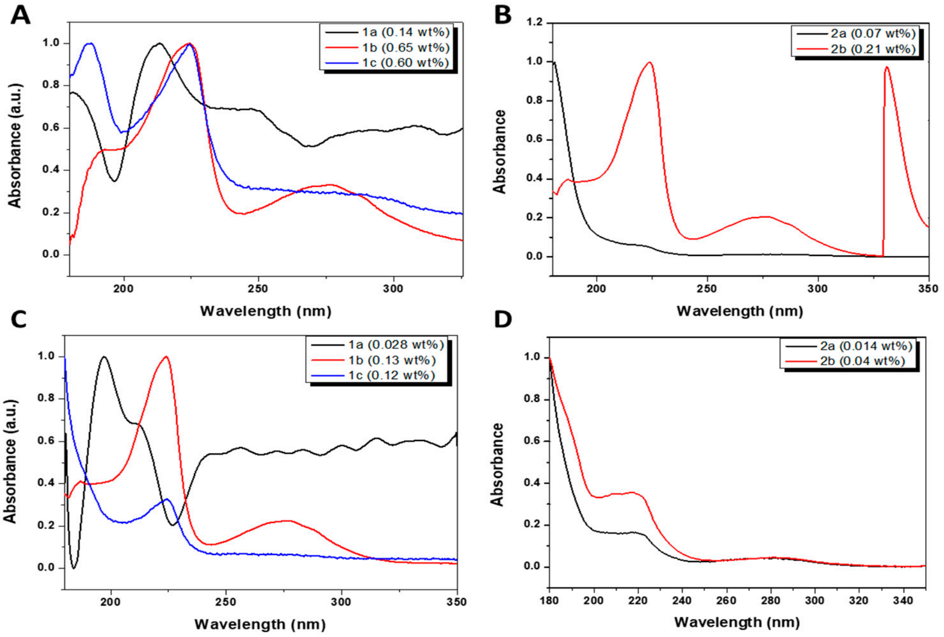

Figure S4. UV-VIS absorption spectra for hydrogelators **1a-c** and **2a,b** in gel phase at the same concentration and pH as that deployed for the CD measurements: A) gels **1a-c;** B) gels **2a,b.** UV-VIS absorption spectra for hydrogelators **1a-c** and **2a,b** in solution, at concentrations well below the CGC: c) gels **1a-c;** D) gels **2a,b.**

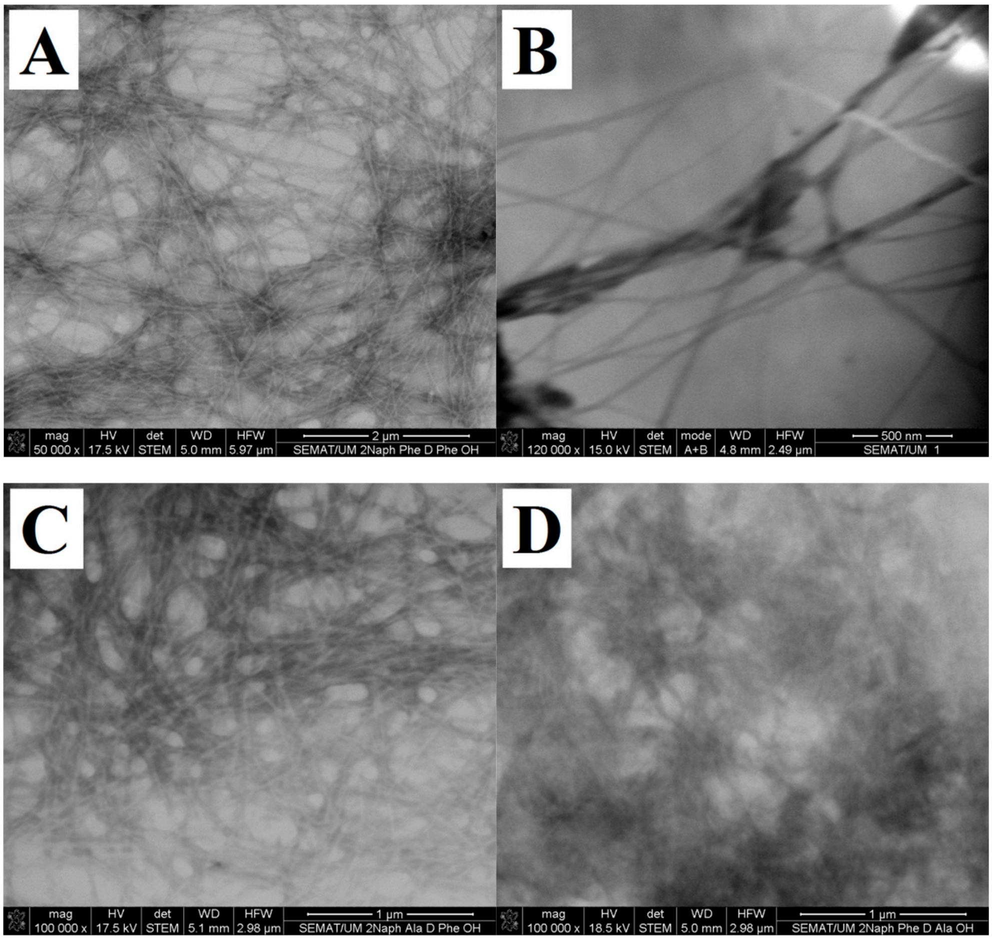

Figure S5. STEM images: A,B) **1a** (gel at 0.5 wt% in phosphate buffer, pH 8), scale A) 2 µm, B) 500 nm; C) **1b** [gel at 0.77 wt%] scale 1 µm; D) **1c** [gel at 0.6 wt%], scale 1 µm.

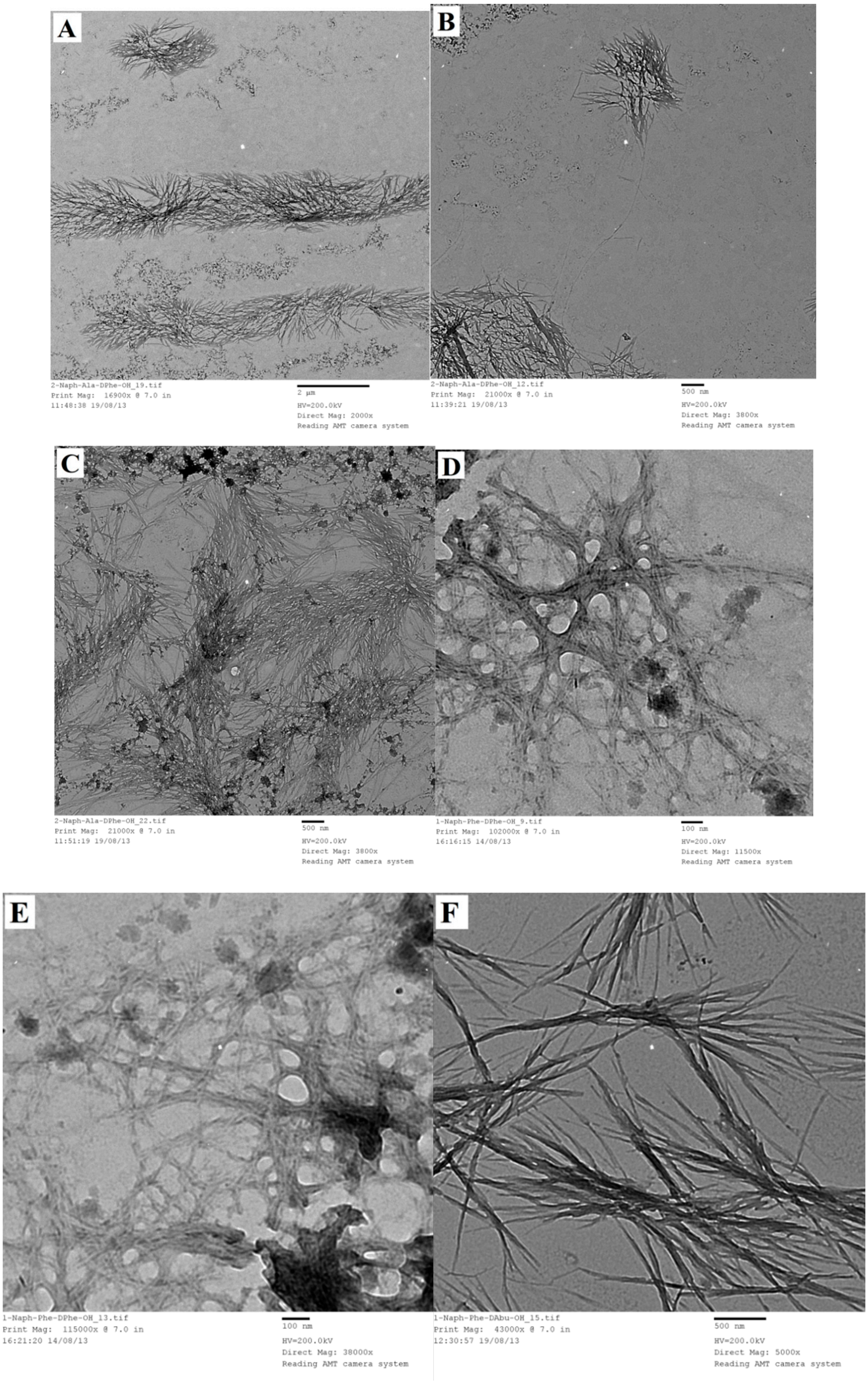

Figure S6. TEM images: A,B,C) **1b** (0.65 wt%), scale A) 2 µm; B,C) 500 nm; D,E) **2a** (0.014 wt%), scale 100 nm; F) **2b** (0.040 wt%), scale 500 nm.

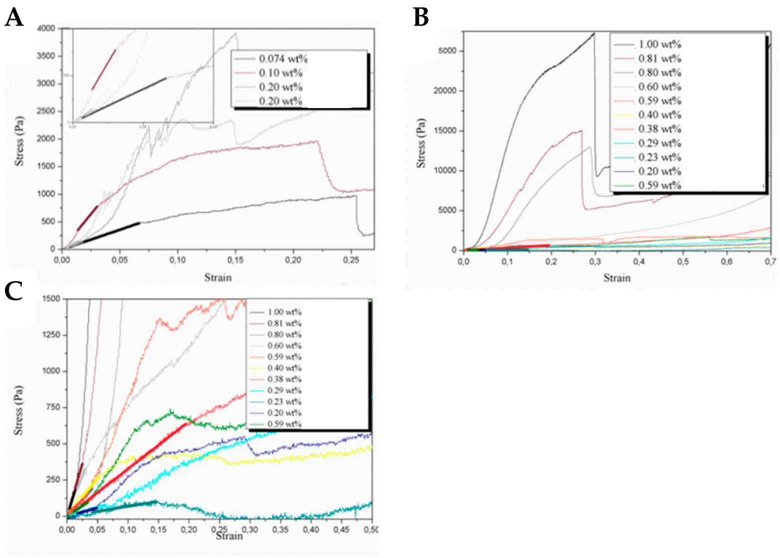

Figure S7. Stress-strain response curves for penetration tests for hydrogels at 25 μm s^{-1} plunger speed.: A) gel **2a;** B) gel **2b**; C) Zoom of the linear regime of the stress-strain response curve of compounds **2b**.
